# A fluorenylidene-acridane that becomes dark in color upon grinding – ground state mechanochromism by conformational change†
†Electronic supplementary information (ESI) available: X-ray crystallographic data for **5** and **6a**, UV-vis light absorption spectra for **6b** and **c**, fluorescence and IR spectra for **6a**, theoretical calculation data (HOMO and LUMO and light absorption) for FAs, thermal analysis data for **6a–d**, powder X-ray diffraction data for **6a–d**, high-pressure X-ray single crystal analysis data (4.6–3.9 GPa) for **6a**, cyclic voltammetry for **6a** and **d**, and carrier transport measurement data for **6a** and **d**. CCDC 1457735–1457738. For ESI and crystallographic data in CIF or other electronic format see DOI: 10.1039/c7sc03567e


**DOI:** 10.1039/c7sc03567e

**Published:** 2017-11-14

**Authors:** Tsuyoshi Suzuki, Hiroshi Okada, Takafumi Nakagawa, Kazuki Komatsu, Chikako Fujimoto, Hiroyuki Kagi, Yutaka Matsuo

**Affiliations:** a Department of Chemistry , School of Science , The University of Tokyo , 7-3-1 Hongo, Bunkyo-ku , Tokyo 113-0033 , Japan; b Department of Mechanical Engineering , School of Engineering , The University of Tokyo , 7-3-1 Hongo, Bunkyo-ku , Tokyo 113-8565 , Japan . Email: matsuo@photon.t.u-tokyo.ac.jp; c Geochemical Research Center , Graduate School of Science , The University of Tokyo , 7-3-1 Hongo, Bunkyo-ku , Tokyo 113-0033 , Japan; d Hefei National Laboratory for Physical Science at the Microscale , University of Science and Technology of China , Hefei , Anhui 230026 , China

## Abstract

We report mechanochromic color change controlled by conformational change (between folded and twisted conformers) of fluorenylidene-acridanes (FAs). FAs with four *N*-alkyl groups (methyl, ethyl, *n*-butyl and *n*-octyl) were synthesized *via* the Barton–Kellogg reaction of diazofluorene and electrophilic *N-tert*-butoxy carbonyl thioacridone, deprotection of the *tert*-butoxy carbonyl group gives fluorenylacridine, and alkylation on the nitrogen atom is done using alkyl tosylate or triflate. FAs were characterized by NMR, UV-vis absorption and photoluminescence spectroscopy, theoretical calculation, cyclic voltammetry, and powder and single-crystal X-ray analyses. The color and folded/twisted conformation of the FAs were changed by the choice of substituent on the nitrogen atom, physical state (solution or solid), and morphology (crystalline or amorphous). Grinding of *N*-methyl FA solids, using an agate mortar, caused the morphology to change from a crystalline to amorphous state, which induced a conformational change from the folded to the twisted conformer, and a mechanochromic color change from yellow to dark green. The reverse color change, along with a morphological and conformational change to the folded conformer, was performed by solvent vapor exposure (chloroform). The twisted and folded conformers showed ambipolar (hole/electron) and hole-only transport properties, respectively.

## Abstract 2

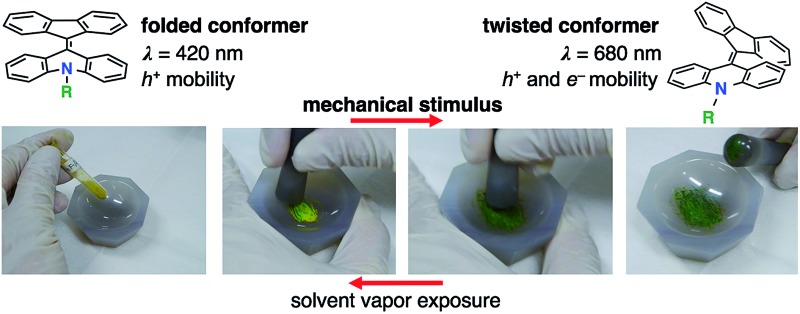
We report mechanochromic color change controlled by conformational change (folded and twisted conformers) of fluorenylidene-acridanes (FAs).

## Introduction

Controlling the optoelectronic properties of organic molecules using external stimuli has received increasing attention from the materials chemistry community and industry owing to their imaging, sensing and memory applications.^1^–^4^ Among such chromic materials, mechanochromic and mechanofluorochromic compounds have received special attention, in regard to converting macroscopic stress into changing molecular properties.^5^–^14^ Much of the mechanochromic behavior is generally induced by a change in the intermolecular π–π stacking structures through crystalline/amorphous morphological change^7^–^12^ and bond formation/cleavage.^1^,^13^,^14^ Compared with these typical examples, there are fewer examples of mechanochromism induced by molecular conformational change. One related example could be tetraphenylethene derivatives showing mechanochromic behavior controlled by changing the dihedral angles among the aromatic groups in the molecules.^15^,^16^

**Fig. 1:**
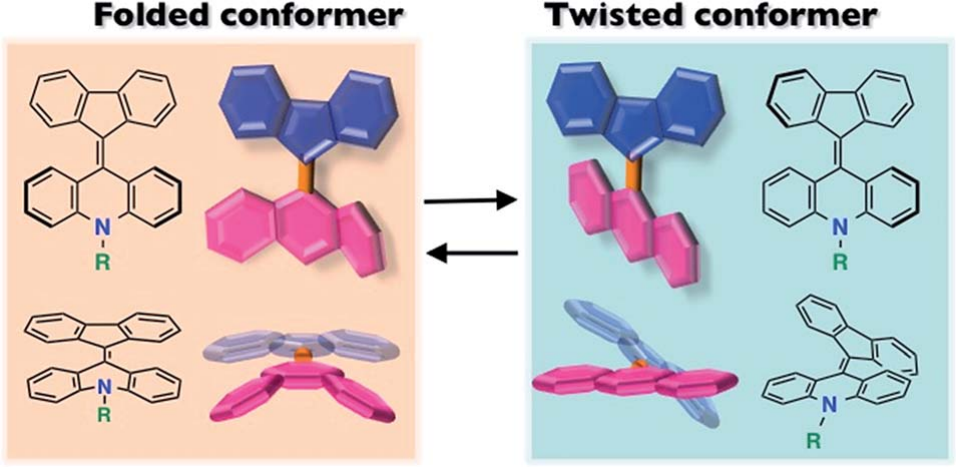
Fluorenylidene-acridanes (FAs) with folded and twisted conformers. The two conformers are in equilibrium in solution.

Overcrowded alkenes, bis(tricyclic) aromatic enes (BAEs), which were reported over 100 years ago,^17^–^20^ show a clear conformational change. Because of steric effects around the central double bond, BAEs possess two conformational isomers, the folded and twisted conformers (Fig. 1). The extent of the π-electron conjugated system differs between these conformers, leading to color changes. Although the photochromism and thermochromism of BAEs have been well demonstrated,^20^–^22^ their mechanochromism has been seldom investigated so far.^18^,^23^ In addition, little attention has been paid to the carrier transport properties of chromic compounds.^4^,^11^,^15^,^24^–^26^

In this work, we report fluorenylidene-acridanes (FAs), a sort of BAE derivative that show color changes that are governed by a folded/twisted conformational change, derived from morphological change caused by mechanical stimulus. FA is composed of fluorene and acridane linked by a double bond. Substituents can be installed on the nitrogen atom of FA to modify its aggregated structure in the solid state. This ability to exploit substituents with ease is a notable difference from typical BAE compounds, such as the oxygen analogue of FA, fluorenylidene-xanthone.^20^,^27^–^30^ We also investigated the carrier transport properties of both isomers in addition to the mechanochromism, to understand the potential mechanical/optical/electronic input/output relationship of this compound.

## Results and discussion

### Synthesis and structural characterization of FAs

**Fig. 2:**
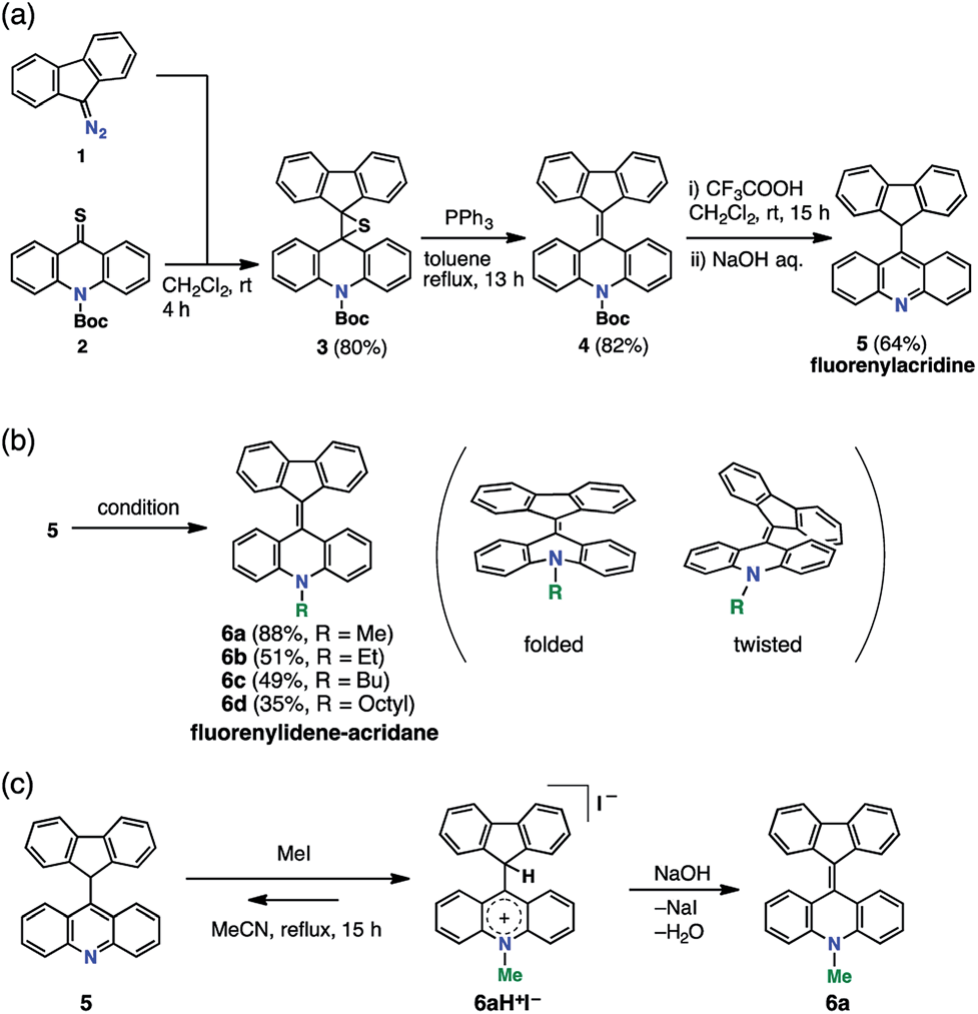
Synthesis of FAs. (a) Barton–Kellogg reaction giving fluorenylacridine. (b) Installation of alkyl chains giving FA derivatives. Conditions: MeOTs, K_2_CO_3_, MeCN, reflux, 15 h (**6a**), EtOTf, K_2_CO_3_, CH_2_Cl_2_, rt, 15 h (**6b**), BuOTf, K_2_CO_3_, CH_2_Cl_2_, rt, 15 h (**6c**), and (i) OctylOH, TfOTf, EtOTs, K_2_CO_3_, *o*-dichlorobenzene, 0 °C, 3 h, (ii) **5**, 50 °C, 17 h (**6d**). (c) Equilibrium between **5** and an acridinium salt (**6aH^+^I^–^**) in the methylation reaction.

**Fig. 3:**
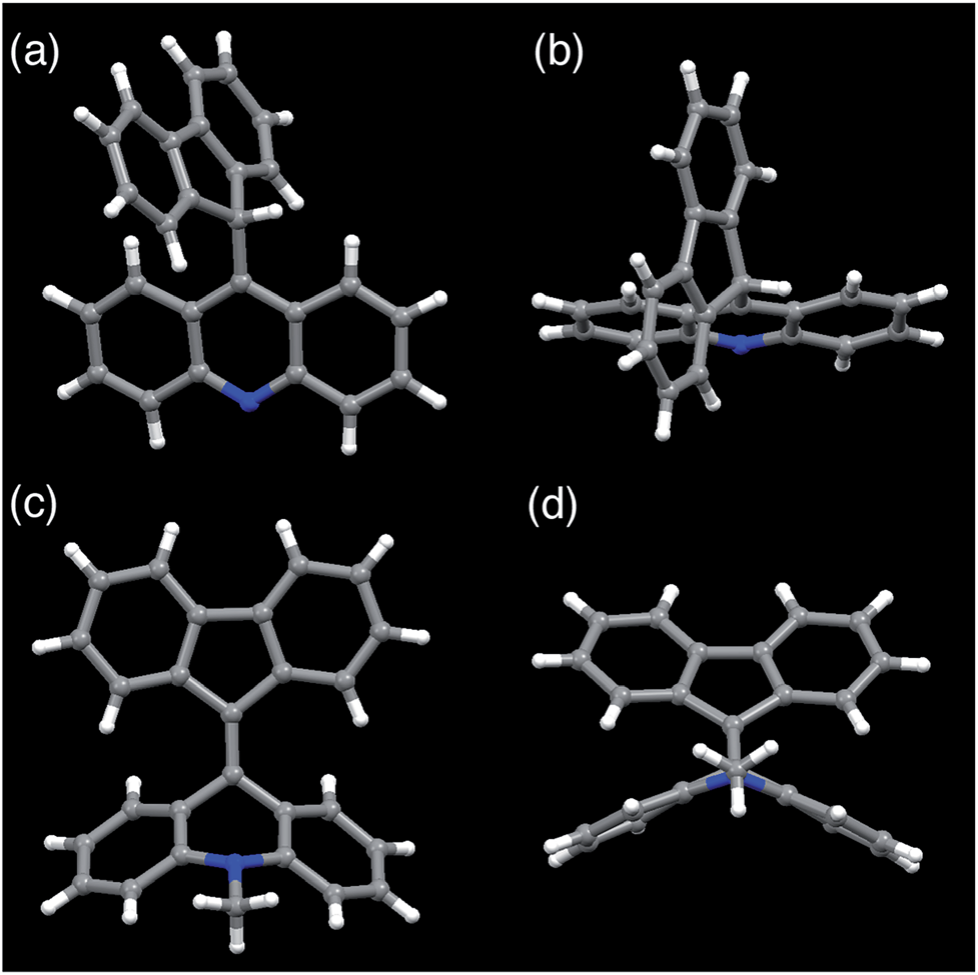
Crystal structures of fluorenylacridine (**5**) and fluorenylidene-acridane (**6a**). (a) Front view of **5**. (b) View from the top of **5**. (c) Front view of **6a**. (d) A folded structure with a view from the bottom of **6a**.

FAs were synthesized from thioacridone and diazofluorene; the Barton–Kellogg reaction was a key step in the synthesis. We introduced an electron-withdrawing *t*-butoxy carbonyl (Boc) group to thioacridone to enhance the electrophilicity of this substrate. *N*-Boc-thioacridone (**2**) successfully reacted with diazofluorene (**1**) to form thiirane compound **3** (Fig. 2a). Addition of triphenylphosphine removed the bridging sulfur atom, and the resulting compound **4** was deprotected by trifluoroacetic acid. Upon deprotection, the hydrogen atom on the nitrogen atom instantly rearranged to form an acridine moiety, giving fluorenylacridine (**5**). The rearranged proton was observed at 6.45 ppm in the ^1^H NMR spectrum, together with eight asymmetric proton peaks corresponding to the acridine moiety, indicating the slow rotation of the acridine moiety on the NMR timescale. The structure of **5** was confirmed by X-ray crystallographic analysis (Fig. 3 and S1†).

Fluorenylacridine (**5**) was used as a precursor to prepare FAs with several different *N*-alkyl chain lengths (Fig. 2b). The treatment of **5** with methyl iodide and quenching with NaOH produced *N*-methyl FA (**6a**). In this reaction, **5** was not completely consumed even in the presence of excess methyl iodide. This result suggested that **5** was in equilibrium with an acridinium salt (**6aH^+^I^–^**)(Fig. 2c). The reverse reaction was suppressed by using potassium carbonate as a base. When methyltosylate was used for the methylation instead of methyl iodide, the yield improved to 88%. To obtain FAs with longer alkyl chains (**6b–d**), the use of alkyl triflates was required because alkyl tosylates were insufficiently electrophilic. The FAs were roughly purified by alumina column chromatography, and then further purified by sublimation under reduced pressure to remove all solvent and trace impurities. FAs were characterized by ^1^H NMR, ^13^C NMR, mass spectrometry, elemental analysis, UV-vis absorption spectroscopy, and X-ray diffraction (XRD) analysis.

Among the FA derivatives, **6a** had exceptionally high crystallinity. It gave single crystals of one polymorph in polar dichloromethane/*n*-hexane solvent and another polymorph in nonpolar benzene/*n*-hexane solvent. These polymorphs were named polymorph 1 and polymorph 2, respectively. Both structures were determined by single-crystal XRD, revealing that both the polymorphs contained folded conformers with similar bond lengths and bond angles (Fig. 3, S2, S3 and Table S1–S4†). Hereafter the data for polymorph 1 are shown and discussed.

### Spectroscopic appearance and morphology of FAs

**Fig. 4:**
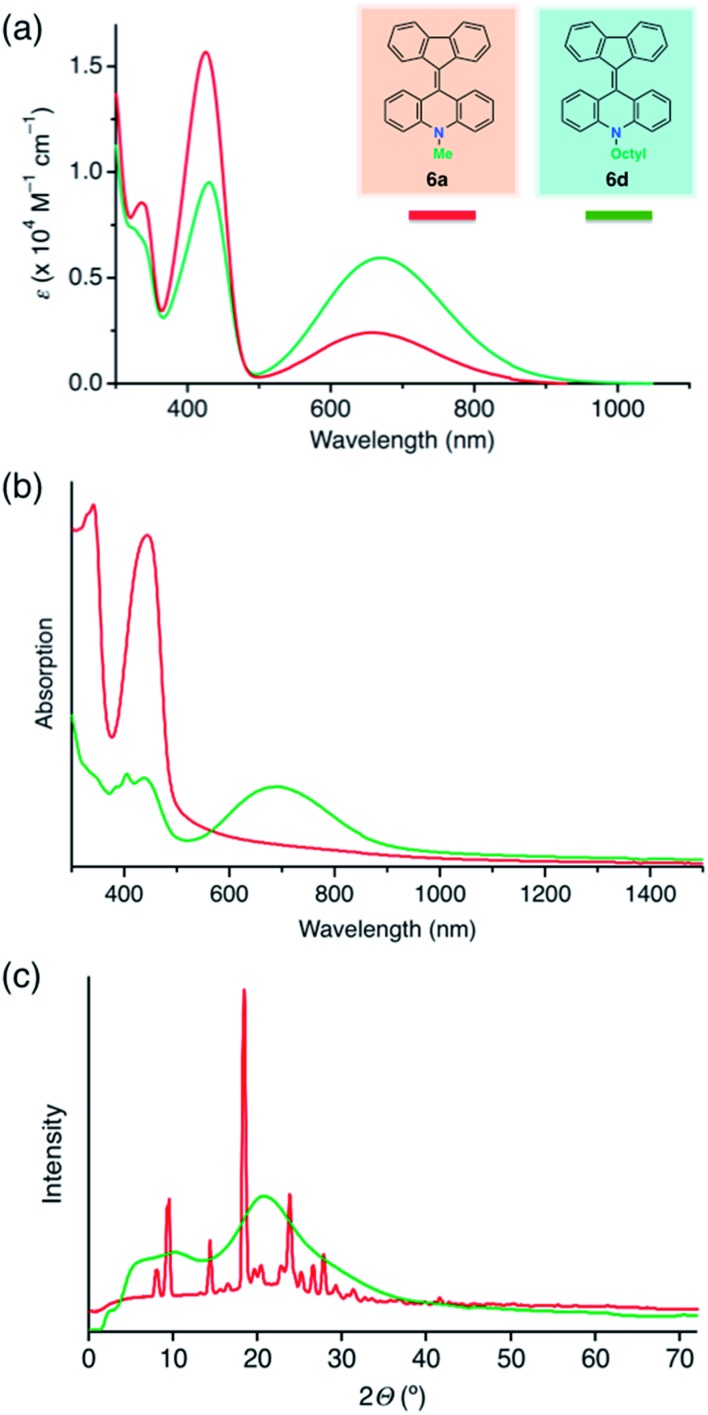
Light absorption spectra and powder XRD patterns of FAs (Red line, **6a**; green line, **6d**). (a) UV-vis absorption spectra in the solution state. The spectra of **6b** and **6c** (Fig. S4†) were similar to that of **6d**. (b) UV-vis absorption spectra in the solid state. The solid films were prepared by spin-coating at 1000 rpm from PhCl solution (10 wt%). (c) The XRD patterns of reprecipitated powder samples.

Compound **6a** formed yellow crystalline solids, but showed a green color in the solution state. In the light absorption spectra, **6a** showed absorption maxima at 420 and 680 nm in dichloromethane (Fig. 4a, red line). We assigned the shorter and longer wavelength absorptions to the folded and twisted conformers in solution, respectively, because a yellow solid of **6a** showing a short wavelength absorption (Fig. 4b, red line) was characterized as the folded conformer by the single-crystal XRD study. We conducted density functional theory (DFT) calculations considering solvent effects with the polarizable continuum model, and found that the folded conformer of **6a** has a slightly lower Gibbs energy, meaning that it is slightly more stable than the twisted conformer (Table S5†). In addition, time-dependent DFT calculations indicated that the folded conformer has a maximum light absorption at 431 nm and a shorter wavelength than that of the twisted conformer (625 nm) (Table S6 and Fig. S6†). These experimental and computational results suggested that the solution of **6a** contained both the conformers in solution. For a related compound, fluorenylidene-xanthone, the light absorption spectra of both the twisted and folded conformers have been recorded by encapsulating the molecule in a self-assembled coordination cage.^30^ Very weak photoluminescence, with a maximum at 530 nm, was detected when a solution of **6a** in dichlorobenzene was excited at a wavelength of 420 nm, while no emission was observed with excitation at 680 nm (Fig. S5a†). Aggregation-induced emission of **6a** was also observed (Fig. S5b†). The fluorescence quantum yield of a microcrystalline powder of **6a** was ∼3%, while those of ground powders and solution phase samples were ∼1% and <1%, respectively. Regarding solid state samples of **6a**, the emission intensity decreased after grinding, and increased after solvent vapor exposure (chloroform) (Fig. S5c†). The solid state emission of amorphous **6b** could not be detected.

Compounds **6b–d** showed different appearances compared to **6a**. In the solution state, all the FAs (**6a–d**) had similar green colors but differences were evident in the UV-vis spectra, with **6b–d** showing higher absorption intensity at 680 nm and lower intensity at 420 nm in comparison with **6a** (Fig. 4a, green line). These differences between **6a** and **6b–d** in solution were also supported by the DFT analysis. Compound **6a** favored the folded conformer, while **6b–d** favored the twisted conformer, which had a slightly lower Gibbs energy than the folded conformer. Accordingly, we surmised that the differences in the light absorption spectra among the FAs were a result of the higher ratio of the twisted conformer in solution for **6b–d** compared to **6a**.

The broad absorption band around 680 nm in solution was common to all the FAs, suggesting it results from the twisted form. By DFT analysis, the longer wavelength light absorption was assigned to the HOMO–LUMO transition of the twisted conformer. The folded conformer has a wider HOMO–LUMO gap, which can be explained by the π-conjugated system of the LUMO being disconnected at the central double bond (Fig. S7†). On the other hand, the twisted conformer has an efficiently conjugated π-system. According to electrostatic potential maps, the higher color contrast in the twisted conformer than in the folded conformer clearly indicates a greater bias in the charge distribution, suggesting a weak charge transfer ability in the twisted conformer (Fig. S8†).

The color difference was much more apparent in the solid state than in solution. In the case of **6a**, yellow block crystals were regularly obtained, but **6b–d** formed green solids. The UV-vis spectra of thin films of **6a** contained a peak at 440 nm without an absorption band in the longer wavelength region (Fig. 4b, red line). In contrast, a broad peak at 700 nm was common to thin films of **6b–d** (Fig. 4b, green line). Because of the similarities of the spectra, we assigned the peaks at 440 and 700 nm to the folded and twisted conformers also in solid state, respectively. Thus, the yellow **6a** film contained only the folded conformer, while the green **6b–d** films contained both the folded and twisted conformers.

We assumed that the different colors of the thin films are related to differences between the film morphologies of **6a** and **6b–d**. According to powder XRD and differential scanning calorimetry measurements of powder samples of FAs, **6b–d** exhibited broadened XRD peaks (Fig. 4c, green line; Fig. S10†) and a glass transition (Fig. S9†), whereas **6a** had a sharp diffraction pattern (Fig. 4c, red line) and no glass transition. These data suggest that the **6a** and **6b–d** solids were in the crystalline and amorphous form, respectively. Notably, **6a** and **6b** had different crystallinity despite a small difference in the length of the *N*-alkyl substituent. The high crystallinity of **6a** comes from the strong packing forces in the crystal and the predominance of the folded conformer in solution. On the other hand, the folded and twisted conformers of **6b–d** are present in solution in a nearly 1 : 1 absorbance ratio, hindering the crystallization of the two conformers to give amorphous mixtures. In the DFT calculations, the length of the alkyl chain had only a small influence on the HOMO and LUMO levels (Table S5†). This means that the differences in color arose from the steric effects of the chains rather than from alteration of the electronic structure. In particular, steric effects led to different stable conformations when the *N*-alkyl substituent was a methyl group and when it was a longer alkyl chain.

### Conformational change-based mechanochromism and carrier transport properties of FAs

**Fig. 5:**
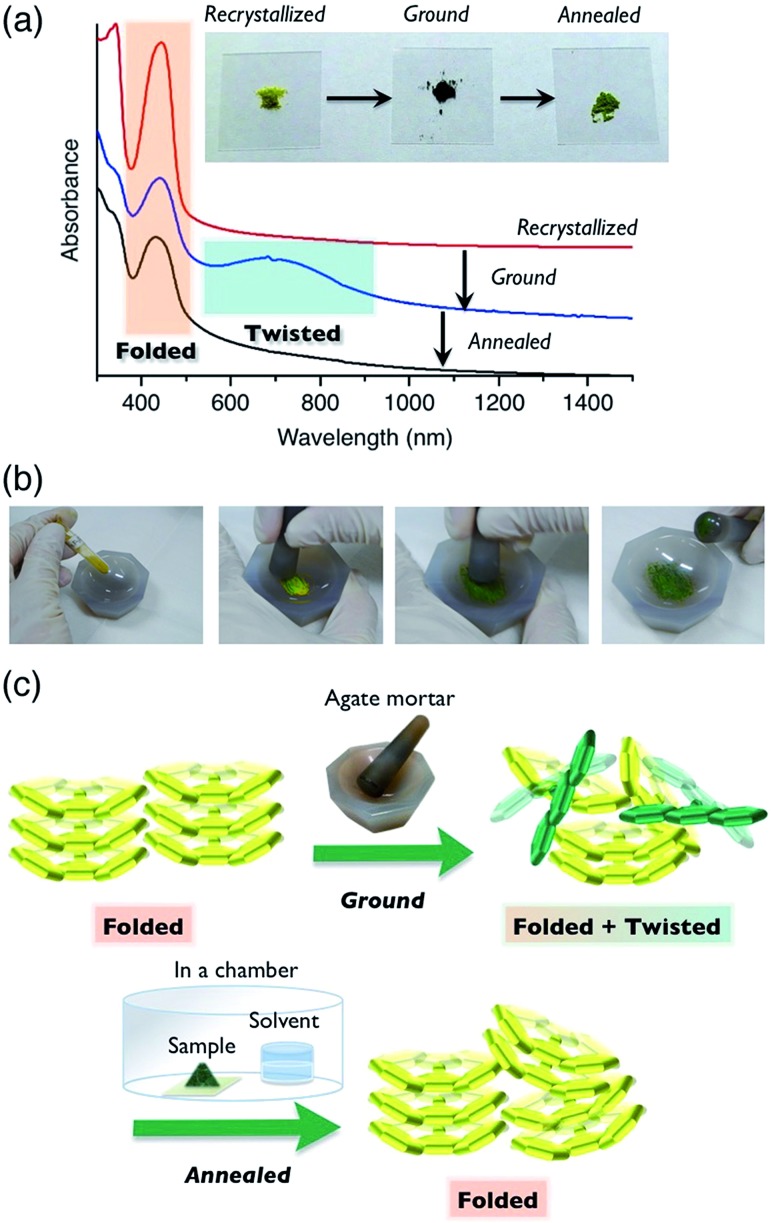
Mechanical-stimulus-driven conformation and color change of **6a**, and its characterization. (a) UV-vis-NIR absorption spectra of **6a** as a thin film on a quartz plate. The spectra for the as-cast film (red), ground film (blue), and film exposed to CHCl_3_ vapor (solvent anneal, black). Inset: photographs of powder samples of **6a** in each state. (b) Grinding **6a** to show ground state mechanochromism. (c) Schematic images of FA molecules in the solid phase.

Compound **6a** showed color switching behavior when it was ground in an agate mortar, changing from yellow to dark green (almost black appearance) (Fig. 5, also see a movie in ESI†). With crushing, the pale-colored crystals became dark green in color. This unique pale-to-dark color change is based on the conformational change of BAEs caused by grinding (Fig. 5b), compared to the colour change caused by hydrostatic pressure using anvils that has previously been discussed.^31^,^32^ The color change was reversed by exposure to chloroform vapor for 5 min. UV-vis absorption spectroscopy was used to monitor the color change (Fig. 5a). A broad peak at 700 nm appeared upon mechanical grinding and disappeared upon exposure to chloroform vapor (solvent anneal). From these results, we assumed that ground **6a** had a solid phase similar to that of **6b–d**, *i.e.* the amorphous phase. The color change was further explored by powder XRD (Fig. S11†). The as-prepared yellow solid of **6a** gave a diffraction pattern that was the same as the one simulated from the single-crystal data. The green powder prepared by grinding gave a weak, broad diffraction pattern, indicating a mixture of amorphous and crystalline domains (Fig. 5c). Exposing the powder to chloroform vapor restored the sharp diffraction pattern. Changes to the IR absorption before and after grinding were not obvious, but there was a small difference (Fig. S12†).

The color change of BAEs (such as bianthrone) induced by high hydrostatic pressure has been seen,^31^ while the color change of BAEs induced by grinding has only briefly been discussed.^18^ Here, we propose two hypotheses based on our results: (i) pressure applied during grinding of the solids changes the conformation from folded to twisted (piezochromism), and (ii) the conformational change from folded to twisted occurs in the amorphous domain of the solid through an equilibrium between the conformers. Even when a high pressure was applied to a single crystal of **6a**, the folded conformer remained unchanged (Fig. S13 and S14 and Table S7†). Similar results were reported for other BAEs.^31^ Accordingly, we concluded that hypothesis (ii), the conformational change induced by a morphological change from a crystalline to amorphous state, is the right phenomenon. Compound **6a** changed from a pale color in the crystalline state to a deep color in the amorphous state. This unique optical characteristic of **6a** marks a contrast with typical organic π-electron conjugated materials, which undergo color change to a slightly pale color when they are ground.

Cyclic voltammetry measurements of **6a** and **6d** showed reversible one-electron reduction and reversible one-electron oxidation processes (Fig. S15 and S16†). They showed a first oxidation corresponding to the acridane moiety at around –0.2 V and a first reduction corresponding to the fluorenylidene moiety at around –1.8 V. In solution, FAs were in equilibrium between the folded and twisted conformers. The solutions contained a mixture of two redox-active molecules. Because the twisted conformers of the FA cation and anion are much more stable than the folded conformers, both the observed first-oxidation and first-reduction potentials mainly consisted of the oxidation and reduction processes for the twisted conformers (see the ESI†for details).^33^ These electrochemical data promised an ambipolar carrier transport ability in the twisted conformer.

**Fig. 6:**
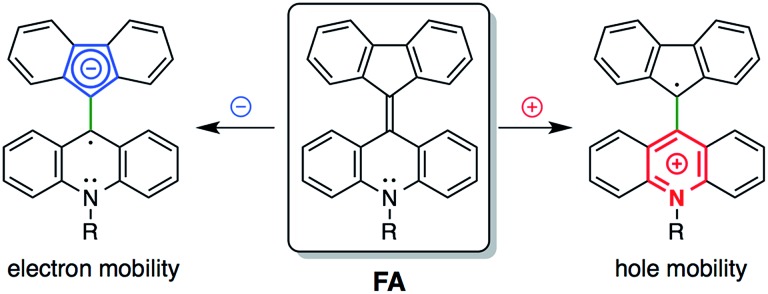
The ambipolar properties of FA.

The carrier transport properties of thin films of the FAs were evaluated by space charge limited current (SCLC) measurements. A few BAEs, such as fluorenyl fluorene as an electron acceptor, have been used in organic electronic devices.^34^–^36^ Compared with fluorenylidene-xanthone, FAs have advantages for hole transport because of their electron-donating nitrogen-containing acridane substructure. Compounds **6a** and **6d** showed hole mobilities of 3.0 × 10^–5^ and 8.4 × 10^–4^ cm^2^ V^–1^ s^–1^, respectively, and compound **6d** in particular additionally showed an electron mobility of 4.8 × 10^–5^ cm^2^ V^–1^ s^–1^ (Fig. 6 and S17†). Thin films of **6a** were crystalline as described above, and contained the folded conformer and showed only hole mobility. We failed to measure amorphous **6a** because we could not grind **6a** films in the devices. As shown by DFT studies (Table S5†), the twisted conformer has higher HOMO and lower LUMO levels than the folded conformer. As a result, compound **6d** has a LUMO level that is deep enough to allow electron mobility. Furthermore, the twisted structure appears to have the advantage of efficient overlap between the molecular orbitals of neighboring molecules. We expect that FAs and their analogues, that have extensive π-conjugated systems, can be applied to future mechanochromic/electronic devices by interchanging mechanical, optical and electronic information.

## Concluding remarks

In summary, we synthesized fluorenylidene-acridanes (FAs), a kind of overcrowded alkene BAE, by connecting the π-conjugated systems of fluorenes and acridanes with a double bond, utilizing the Barton–Kellogg reaction. We expected a large advantage in the design of this molecule because of the nitrogen atom in FAs, compared with the related compound fluorenylidene-xanthone, which contains an oxygen atom. The nitrogen atom enables the installation of an *N*-alkyl group for controlling the morphology in the solid state, and adding hole transport properties, which can open an avenue for applications in organic electronics. The hybrid donor (acridane) and acceptor (fluorene) structure connected with a double bond is also advantageous as it has a small frontier orbital energy gap for long-wavelength light absorption and ambipolar carrier transport properties.

FAs **6a–d** with different *N*-alkyl chain lengths were found to exhibit different colors in solution and in the solid state. We observed a large difference between the light absorption spectra of compound **6a** and compounds **6b–d** in solution. The existence of a β-carbon in the *N*-alkyl substituent played a critical role in changing the equilibrium between the folded and twisted conformers even in the solution state. With ethyl, *n*-butyl and *n*-octyl substituents, the amount of twisted conformer increased. Furthermore, in the solid state, differences in the alkyl chain length induced differences in the crystallinity, which affected the ratio of the folded and twisted conformers. A clear difference in morphology was also observed between a methyl group and longer alkyl chains. Compound **6a** was highly crystalline, and contained the folded conformer in the crystalline form, which was characterized with X-ray crystallography. On the other hand, compounds **6b–d** gave amorphous solids containing both the twisted and folded conformers.

By applying a mechanical stimulus to the solids or thin films of FAs, **6a** exhibited mechanochromic behavior by a transition between the crystalline and amorphous phases. This transition led to packing structure and conformational changes, and thus a color change. Grinding **6a** changed the morphology from crystalline to amorphous, which induced a conformational change from the folded to the twisted conformer, giving a mechanochromic color change from yellow to dark green. This phenomenon, the crushing of crystals leading to a dark color, is quite unique, because in ordinary organic compounds grinding the solid makes their color pale. A reverse color change from dark green to yellow occurred with solvent vapor exposure leading to a conformational change to the folded conformer. The present work provides an obvious example of the ground state mechanochromism by hand grinding, with drastic color change.

Carrier transport experiments elucidated the electronic properties of FAs. Amorphous thin films of **6d** containing the twisted conformer showed ambipolar carrier transport properties with a hole mobility of 8.4 × 10^–4^ cm^2^ V^–1^ s^–1^ and an electron mobility of 4.8 × 10^–5^ cm^2^ V^–1^ s^–1^. On the other hand, crystalline thin films of **6a** consisted of the folded conformer and exhibited only hole transfer properties (3.0 × 10^–5^ cm^2^ V^–1^ s^–1^). One possible future application of FAs would be in color-changing mechanical sensors for detecting mechanical stimuli and outputting an electric signal.

## Experimental

### General

All NMR spectra were recorded at 400 MHz (JEOL ECA-500 spectrometer) or 500 MHz (Bruker AVANCE III 500 spectrometer). NMR spectra were recorded in parts per million (ppm, *δ* scale) from the residual protons of CDCl_3_ for ^1^H NMR (*δ* 7.26 ppm for chloroform) and the carbon of CDCl_3_ for ^13^C NMR (*δ* 77.0 ppm). The data are presented as follows: chemical shift, multiplicity (s = singlet, d = doublet, t = triplet, m = multiplet and/or multiplet resonances), coupling constant in hertz (Hz), signal area integration in natural numbers and assignment (italic). Mass spectra were acquired using a Bruker microTOF II (APCI) spectrometer. High-resolution mass spectra were obtained with a calibration standard of polyethylene glycol (MW 600) and elemental analysis was performed at the University of Tokyo, Department of Chemistry, Organic Elemental Analysis Laboratory. IR absorption was measured on a JASCO FT/IR-6100 spectrometer equipped with an attenuated total reflection (ATR) with diamond, and the data are reported as wavenumber in cm^–1^.

### Synthesis of dispiro[*N*-(*tert*-butoxy carbonyl)-acridane-9,2′-thiirane-3′,9′′-fluorene] (**3**)

*N*-(*tert*-Butoxy carbonyl)-9-acridone (4.92 g, 15.8 mmol) and 9-diazofluorene (3.05 g, 15.9 mmol, 1.1 equiv.) were mixed in dichloromethane (160 mL, 0.10 M) for 4 h. After drying, the crude solids were purified by silica gel column chromatography with chloroform/hexane (1 : 1) to obtain yellow solids (6.02 g, 80%). ^1^H NMR (400 MHz, CDCl_3_): *δ* = 7.87 (dd, *J* = 7.6, 1.6 Hz, 2H), 7.52 (d, *J* = 7.8 Hz, 1H), 7.38 (dd, *J* = 8.0, 1.1 Hz, 2H), 7.27–7.15 (m, 6H), 6.86 (td, *J* = 7.6, 0.9 Hz, 2H), 6.78 (d, *J* = 7.8 Hz, 2H), 1.14 (s, 9H). ^13^C{^1^H} NMR (100.53 MHz, CDCl_3_): *δ* = 150.6, 141.7, 140.9, 140.8, 134.0, 127.8, 127.1, 127.0, 126.1, 124.3, 124.2, 124.1, 119.4, 81.0, 57.5, 57.0, 27.6. HR-MS (APCI^+^): calcd. for C_31_H_25_NO_2_S (M)^+^ = 475.1601, found 475.1597. Mp 217.2–220.0 °C (decomposition).

### Synthesis of *N*-(*tert*-butoxy carbonyl)-10-(fluoren-9-ylidene)-acridane (**4**)

Dispiro[*N*-(*tert*-butoxy carbonyl)-acridane-9,2′-thiirane-3′,9′′-fluorene] (5.80 g, 12.2 mmol) and triphenylphosphine (3.20 g, 12.2 mmol, 1.0 equiv.) were refluxed in toluene (61 mL, 0.20 M) for 13 h. The reaction mixture was dried to get yellow crude solids, which were purified by column chromatography and following reprecipitation from chloroform/methanol, the pure compound was obtained as a yellow powder (4.41 g, 82%). ^1^H NMR (400 MHz, CDCl_3_): *δ* = 7.87 (d, *J* = 8.3 Hz, 2H), 7.84 (d, *J* = 7.8 Hz, 4H), 7.69 (d, *J* = 7.3 Hz, 2H), 7.34 (td, *J* = 7.8, 1.5 Hz, 2H), 7.29 (td, *J* = 7.3, 0.9 Hz, 2H), 7.19 (td, *J* = 7.6, 1.2 Hz, 2H), 7.03 (t, *J* = 7.1 Hz, 2H), 1.59 (s, 9H). ^13^C{^1^H} NMR (100.53 MHz, CDCl_3_): *δ* = 151.7, 140.9, 139.4, 138.2, 133.6, 133.1, 130.8, 128.2, 127.8, 127.6, 125.9, 125.7, 125.1, 124.0, 119.3, 82.4, 28.3. HR-MS (APCI^+^): calcd. for C_31_H_25_NO_2_ (M)^+^ = 443.1880, found 443.1880. Mp 224.0–226.0 °C (decomposition).

### Synthesis of 9-(9-fluorenyl)-acridine (**5**)

*N*-(*tert*-Butoxy carbonyl)-10-(fluoren-9-ylidene)-acridane (4.20 g, 9.47 mmol) was dissolved in dichloromethane (95 mL, 0.10 M) with trifluoroacetic acid (3.24 g, 2.25 mL, 3.0 equiv.) and stirred for 15 h. After removing the solvent, methanol and NaOH aq. (0.1 M) were added to neutralize the solution. The organic compounds were extracted with dichloromethane and dried with magnesium sulfate. Removing the solvent resulted in yellow crude product, which was purified by silica gel column chromatography. Pale green solids (2.08 g, 64%) were obtained by drying the product. ^1^H NMR (400 MHz, CDCl_3_): *δ* = 8.65 (d, *J* = 9.2 Hz, 1H), 8.44 (d, *J* = 8.7 Hz, 1H), 8.22 (d, *J* = 8.7 Hz, 1H), 7.99 (d, *J* = 7.3 Hz, 2H), 7.87 (ddd, *J* = 8.8, 6.5, 1.0 Hz, 1H), 7.63 (ddd, *J* = 8.8, 6.5, 1.3 Hz, 1H), 7.50 (ddd, *J* = 8.7, 6.4, 1.4 Hz, 1H), 7.44 (t, *J* = 7.6 Hz, 2H), 7.16 (td, *J* = 7.6, 0.9 Hz, 2H), 7.05 (d, *J* = 7.3 Hz, 2H), 6.88 (ddd, *J* = 8.8, 6.5, 1.3 Hz, 1H), 6.77 (d, *J* = 9.2 Hz, 1H), 6.45 (s, 1H). ^13^C{^1^H} NMR (100.53 MHz, CDCl_3_): *δ* = 148.9, 148.8, 143.4, 140.1, 130.7, 130.0, 129.6, 129.4, 127.5, 127.4, 127.1, 126.4, 125.3, 125.1, 124.3, 124.1, 123.6, 120.5, 48.2. MS (APCI^+^) 343.1154. Mp 220.2–223.0 °C (decomposition), anal. calcd. for C_26_H_17_N: C, 90.93; H, 4.99; N, 4.08. Found: C, 90.90; H, 5.04; N, 3.99.

### Synthesis of *N*-methyl-10-(fluoren-9-ylidene)-acridane (**6a**)

In a 30 mL two-neck flask, 9-(9-fluorenyl)-acridine (500 mg, 1.46 mmol), potassium carbonate (2.02 g, 14.6 mmol) and methyl tosylate (971 mg, 5.21 mmol, 3.57 equiv.) were added to acetonitrile (15 mL, 0.10 M). The reaction mixture was refluxed for 15 h. The resulting mixture was poured into water to extract the organic compounds with chloroform. The crude product was further purified by reprecipitation from dichloromethane/methanol. The solids were collected by suction to get greenish yellow solids (459.2 mg, 88%). ^1^H NMR (400 MHz, CDCl_3_): *δ* = 7.95 (dd, *J* = 7.8, 1.4 Hz, 2H), 7.74 (d, *J* = 7.8 Hz, 2H), 7.71 (d, *J* = 7.3 Hz, 2H), 7.41 (t, *J* = 7.1 Hz, 2H), 7.25 (m, 4H), 7.11 (t, *J* = 7.6 Hz, 2H), 7.02 (t, *J* = 7.1 Hz, 2H), 3.59 (s, 3H). ^13^C{^1^H} NMR (100.53 MHz, CDCl_3_): *δ* = 143.9, 140.3, 138.8, 133.8, 129.0, 128.8, 128.7, 127.0, 125.6, 124.7, 124.2, 120.3, 119.1, 113.3, 33.4. MS (APCI^+^) 357.1325. Mp 261.4–262.6 °C, anal. calcd. for C_27_H_19_N: C, 90.72; H, 5.36; N, 3.92. Found: C, 90.64; H, 5.55; N, 3.84.

### Synthesis of *N*-ethyl-10-(fluoren-9-ylidene)-acridane (**6b**)

In a 25 mL Schlenk flask, 9-(9-fluorenyl)-acridine (49.8 mg, 0.145 mmol), potassium carbonate (202.8 mg, 1.47 mmol) and ethyl trifluoromethanesulfonate (*ca.* 0.060 mL, 83.6 mg, 0.469 mmol, 3.2 equiv.) were added to dichloromethane (1.5 mL, 0.1 M). The reaction mixture was refluxed for 13 h. The resulting mixture was poured into water to extract the organic compounds with chloroform. The crude product was purified by alumina column chromatography with an eluent of CHCl_3_/hexane (1 : 2). The solids were further purified by sublimation *in vacuo* to get green solids (27.3 mg, 51%). ^1^H NMR (500 MHz, CDCl_3_): *δ* = 8.12 (dd, *J* = 7.9, 1.6 Hz, 2H), 7.77 (d, *J* = 7.6 Hz, 2H), 7.74 (d, *J* = 8.2 Hz, 2H), 7.41 (ddd, *J* = 8.5, 6.9, 1.6 Hz, 2H), 7.33 (d, *J* = 7.9 Hz, 2H), 7.23 (td, *J* = 7.6, 0.95 Hz, 2H), 7.05 (td, *J* = 6.9, 0.95 Hz, 4H), 4.25 (q, *J* = 7.1 Hz, 2H), 1.55 (t, *J* = 7.1 Hz, 3H). ^13^C{^1^H} NMR (125.76 MHz, CDCl_3_): *δ* = 12.6, 40.8, 113.9, 119.2, 120.5, 123.4, 124.6, 125.4, 125.9, 127.1, 129.4, 130.8, 136.3, 139.2, 139.7, 141.4. HR-MS (APCI^+^): calcd. for C_28_H_21_N (M^+^) = 371.1674, found 371.1664.

### Synthesis of *N*-butyl-10-(fluoren-9-ylidene)-acridane (**6c**)

In a 25 mL Schlenk, 9-(9-fluorenyl)-acridine (100.1 mg, 0.291 mmol), potassium carbonate (405.4 mg, 2.93 mmol) and *n*-butyl trifluoromethanesulfonate (*ca.* 0.160 mL, 197.1 mg, 0.956 mmol, 3.3 equiv.) were added to dichloromethane (3 mL, 0.1 M). The reaction mixture was refluxed for 20.5 h. Then *n*-butyl trifluoromethanesulfonate (*ca.* 0.320 mL, 409.8 mg, 1.99 mmol, 6.8 equiv.) was added into the Schlenk and stirred for an additional 4 h. The resulting mixture was poured into water to extract the organic compounds with chloroform. The crude product was purified by alumina column chromatography with an eluent of CHCl_3_/hexane (1 : 9 to 1 : 2). The solids were further purified by sublimation *in vacuo* to get green solids (57.5 mg, 49%). ^1^H NMR (500 MHz, CDCl_3_): *δ* = 8.10 (dd, *J* = 7.9, 1.3 Hz, 2H), 7.77 (d, *J* = 7.6 Hz, 2H), 7.74 (d, *J* = 7.9 Hz, 2H), 7.41 (ddd, *J* = 8.5, 7.3, 1.6 Hz, 2H), 7.30 (d, *J* = 8.5 Hz, 2H), 7.24 (td, *J* = 7.3, 0.63 Hz, 2H), 7.06 (td, *J* = 7.6, 1.3 Hz, 4H), 4.14 (t, *J* = 7.7 Hz, 2H), 1.94 (quintet, *J* = 7.7 Hz, 2H), 1.53 (m, 2H), 1.02 (t, *J* = 7.4 Hz, 3H). ^13^C{^1^H} NMR (125.76 MHz, CDCl_3_): *δ* = 13.8, 20.2, 29.0, 45.9, 114.1, 119.2, 120.5, 123.5, 124.7, 125.4, 126.0, 127.1, 129.3, 130.6, 136.1, 139.3, 139.7, 141.8. HR-MS (APCI^+^): calcd. for C_30_H_25_N (M^+^) = 399.1987, found 399.1988.

### Synthesis of *N*-octyl-10-(fluoren-9-ylidene)-acridane (**6d**)

In a 50 mL two-neck flask, potassium carbonate (10.1 g, 73.0 mmol) and octanol (569 mg, 0.700 mL, 4.37 mmol, 3.00 equiv.) were dispersed in *o*-dichlorobenzene (22 mL), which was cooled to 0 °C and trifluoromethanesulfonic anhydride (1.41 g, 5.01 mmol, 3.44 equiv.) was slowly added. After stirring for 3 h, 9-(9-fluorenyl)-acridine (500 mg, 1.46 mmol) was added and heated to 50 °C for 17 h. *o*-Dichlorobenzene was distilled to extract the organic compounds with water/chloroform. The evaporation of solvent gave a deep green oil, which was passed through an alumina column and reprecipitated from ether/methanol at a low temperature. A lump of sticky deep green solid was obtained (230.4 mg, 35%). ^1^H NMR (500 MHz, CDCl_3_): *δ* = 8.08 (dd, *J* = 7.9, 1.9 Hz, 2H), 7.75 (ddd, *J* = 7.6, 1.3, 0.6 Hz, 2H), 7.72 (d, *J* = 7.9 Hz, 2H), 7.40 (ddd, *J* = 8.2, 6.9, 1.6 Hz, 2H), 7.29 (d, *J* = 7.6 Hz, 2H), 7.22 (dd, *J* = 7.6, 1.3 Hz, 2H), 7.04 (dddd, *J* = 7.9, 6.9, 2.2, 0.9 Hz, 4H), 4.13 (t, *J* = 7.9 Hz, 2H), 1.94 (q, *J* = 7.6 Hz, 2H), 1.45 (q, *J* = 6.9 Hz, 2H), 1.40–1.34 (m, 2H), 1.28–1.19 (m, 6H), 0.83 (t, *J* = 6.9 Hz, 3H). ^13^C{^1^H} NMR (100.53 MHz, CDCl_3_): *δ* = 141.7, 139.7, 139.2, 136.2, 130.6, 129.3, 127.0, 129.3, 127.0, 125.9, 125.4, 124.7, 123.4, 120.4, 119.2, 114.1, 46.2, 31.7, 29.22, 29.20, 27.0, 26.9, 22.6, 14.0. MS (APCI^+^) 455.2240. Mp: not clear, anal. calcd. for C_34_H_33_N: C, 89.63; H, 7.30; N, 3.07. Found: C, 89.50; H, 7.35; N, 3.00.

### Electrochemical analysis

Cyclic voltammetry (CV) and differential pulse voltammetry (DPV) were performed using a Hokuto Denko HZ-5000 voltammetric analyzer. All CV measurements were carried out under argon gas, in a one-compartment cell equipped with a glassy-carbon working electrode, a platinum wire counter electrode, and an Ag/Ag^+^ reference electrode. The supporting electrolyte was a 0.1 mol L^–1^ dichloromethane solution of tetrabutylammonium hexafluorophosphate (TBAPF_6_).

### X-ray crystallographic analysis

X-ray crystallographic analyses were performed under ambient pressure using a RIGAKU R-AXIS RAPID II (imaging plate detector) with monochromic CuKα (*λ* = 1.5406 Å) radiation. The positional and thermal parameters were refined by a full-matrix least-squares method using the SHELXL97 program on Yadokari-software. Details of the X-ray crystallographic analysis under high pressure are described in the ESI.†

### Computational studies

All calculations were carried out using the Gaussian09 package and the B3LYP functional. The solvent effects were estimated by the polarizable continuum model (PCM) method with the dielectric constant for dichloromethane. A 6-31G(d) basis set was used for each level. The calculation levels are described as “B3LYP/6-31G(d)”.

### SCLC measurements

Hole-only devices were fabricated with a configuration of glass/ITO/PEDOT:PSS/**6a,d**/MoO_3_/Al. PEDOT:PSS layers were formed on the glass/ITO substrate to obtain a 30 nm-thick film. Compounds **6a** and **d** in chlorobenzene were spin-coated, then MoO_3_ (10 nm) was deposited under vacuum (3 × 10^–4^ Pa), and then Al (100 nm) was deposited. The electron-only device configuration was glass/Al/**6a,d**/LiF/Al. After the spin-coating of compounds **6a** and **d** in chlorobenzene, LiF (0.6 nm) and Al (100 nm) were deposited under vacuum. *J*–*V* curves of these devices were measured with a sweeping voltage using a Keithley 2400 source meter unit. The film thicknesses were obtained using a Dektak 6M stylus profiler. The *J*–*V* curves were fit to the equation shown in the ESI† to obtain the hole and electron mobilities.

## Conflicts of interest

There are no conflicts to declare.

## Supplementary Material

Supplementary movie

Supplementary information

Crystal structure data
