# Supplementary material for: A fluorenylidene-acridane that becomes dark in color upon grinding – ground state mechanochromism by conformational change
Source: Chem Sci. 2017 Nov 14;9(2):475–82. doi: 10.1039/c7sc03567e (PMC5868075; doi:10.1039/c7sc03567e)
Supplement: Supplementary file 2 [file SC-009-C7SC03567E-s002.pdf]

Electronic Supplementary Material (ESI) for Chemical Science.
This journal is © The Royal Society of Chemistry 2017

Electronic Supplementary Information

Fluorenylidene-acridane That Becomes Dark Color by Grinding – Ground State Mechanochromism by Conformational Change

Tsuyoshi Suzuki,[1] Hiroshi Okada,[2] Takafumi Nakagawa,[2] Kazuki Komatsu,[3] Chikako Fujimoto,[3] Hiroyuki Kagi[3] and Yutaka Matsuo[2,4*]

[1] *Department of Chemistry, School of Science, The University of Tokyo, 7-3-1 Hongo, Bunkyo-ku, Tokyo 113-0033, Japan*

[2] *Department of Mechanical Engineering, School of Engineering, The University of Tokyo, 7-3-1 Hongo, Bunkyo-ku, Tokyo 113-8656, Japan*

[3] *Geochemical Research Center, Graduate School of Science, The University of Tokyo, 7-3-1 Hongo, Bunkyo-ku, Tokyo 113-0033, Japan*

[4] *University of Science and Technology of China, Hefei, Anhui 230026, China*

** E-mail: matsuo@photon.t.u-tokyo.ac.jp*

Table of Contents:

1. Synthetic Schemes S2
2. X-ray Single Crystal Structures of 5 and 6a S4
3. Light Absorption and Photoluminescence S10
4. DFT Studies S12
5. Thermal Properties S17
6. Powder X-ray Diffraction Analysis S18
7. IR Spectra before and after grinding of 6a S20
8. High-Pressure X-ray Single Crystal Analysis S21
9. Electrochemical Studies S24
10. Carrier Transport Properties S27
11. References S28

1. Synthetic Schemes

Synthesis of 9-fluorenehydrazone

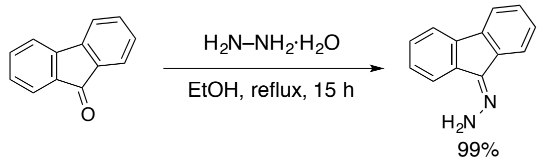

Synthesis of 9-diazofluorene (1)

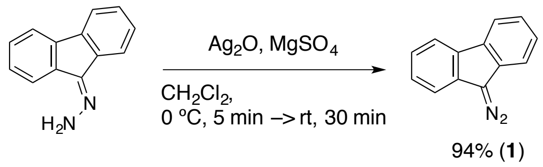

Synthesis of *N*-(*tert*-butoxy carbonyl)-9-acridone

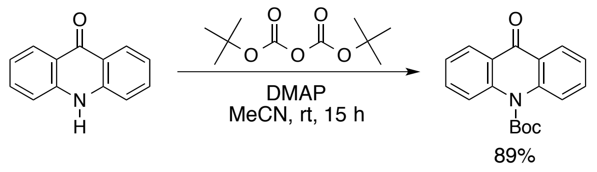

Synthesis of *N*-(*tert*-butoxy carbonyl)-9-thioacridone (2)

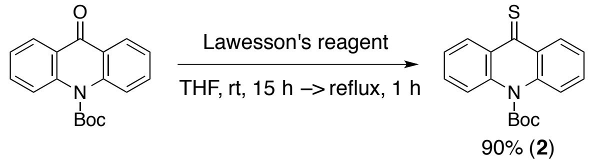

Synthesis of dispiro[*N*-(*tert*-butoxy carbonyl)-acridane-9,2'-thiirane-3',9''-fluorene] (3)

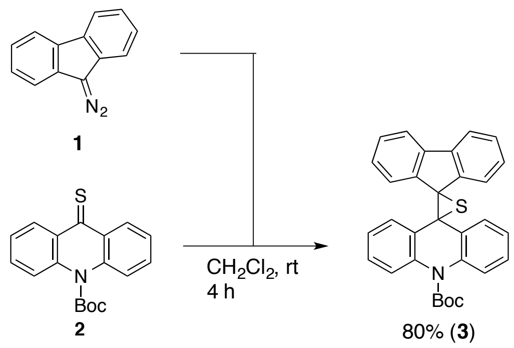

Synthesis of *N*-(*tert*-butoxy carbonyl)-10-(fluoren-9-ylidene)-acridane (4)

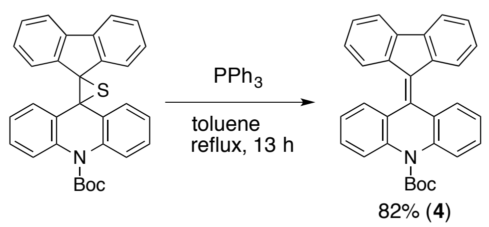

Synthesis of 9-(9-fluorenyl)-acridine (5)

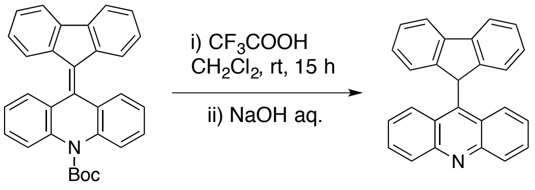

64% (**5**)

Synthesis of *N*-methyl-10-(fluoren-9-ylidene)-acridane (6a)

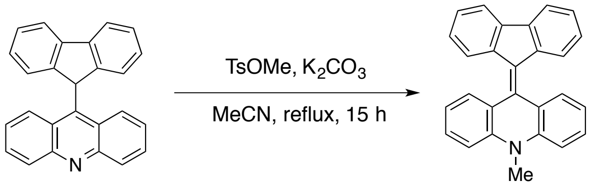

5

88% (**6a**)

Synthesis of *N*-ethyl-10-(fluoren-9-ylidene)-acridane (6b)

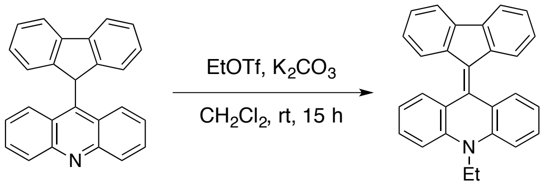

51% (**6b**)

Synthesis of *N*-buthyl-10-(fluoren-9-ylidene)-acridane (6c)

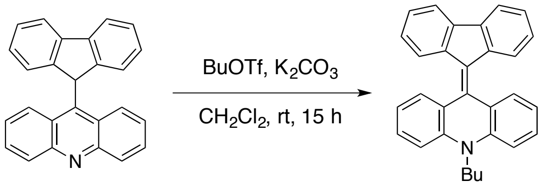

49% (**6c**)

Synthesis of *N*-octyl-10-(fluoren-9-ylidene)-acridane (6d)

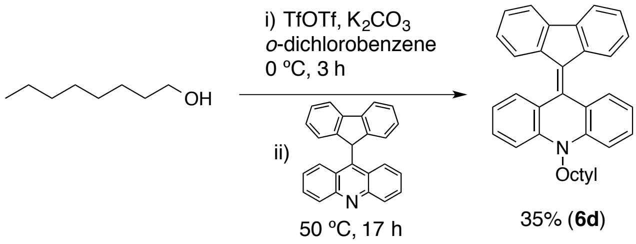

35% (**6d**)

2. X-ray Single Crystal Structures of 5 and 6a

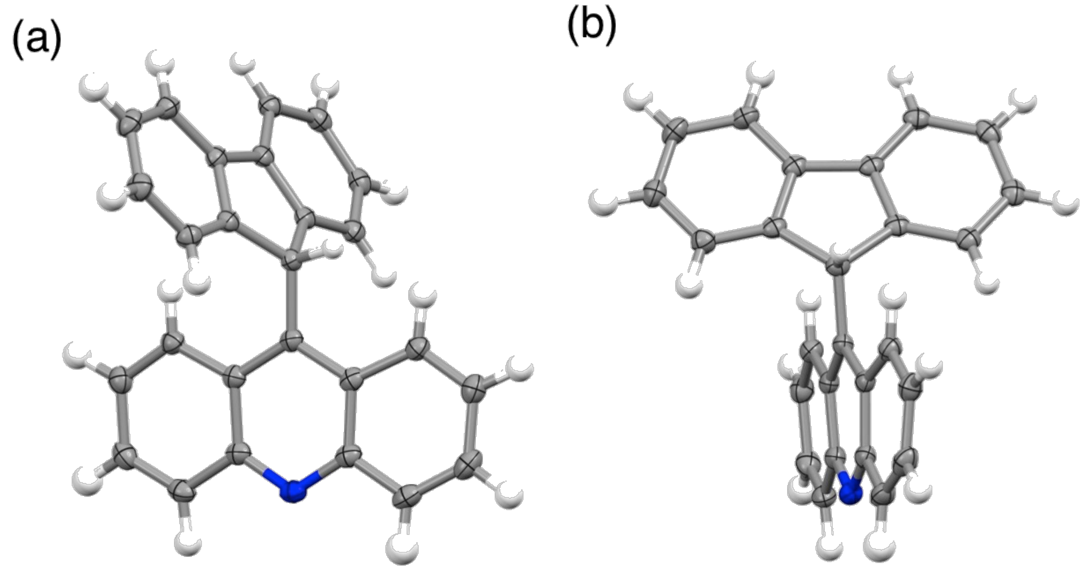

Figure S1. Crystal structures of **5**.

Single crystal structures of **6a** formed polymorph depending on the crystallizing solution system. Polar solvent system like CH_2Cl_2/MeOH, $CHCl_3$/MeOH and toluene/MeOH resulted in **Polymorph 1**. Nonpolar solvent like benzene/hexane gave **Polymorph 2**. Both structures involved quite similar structures of **6a**.

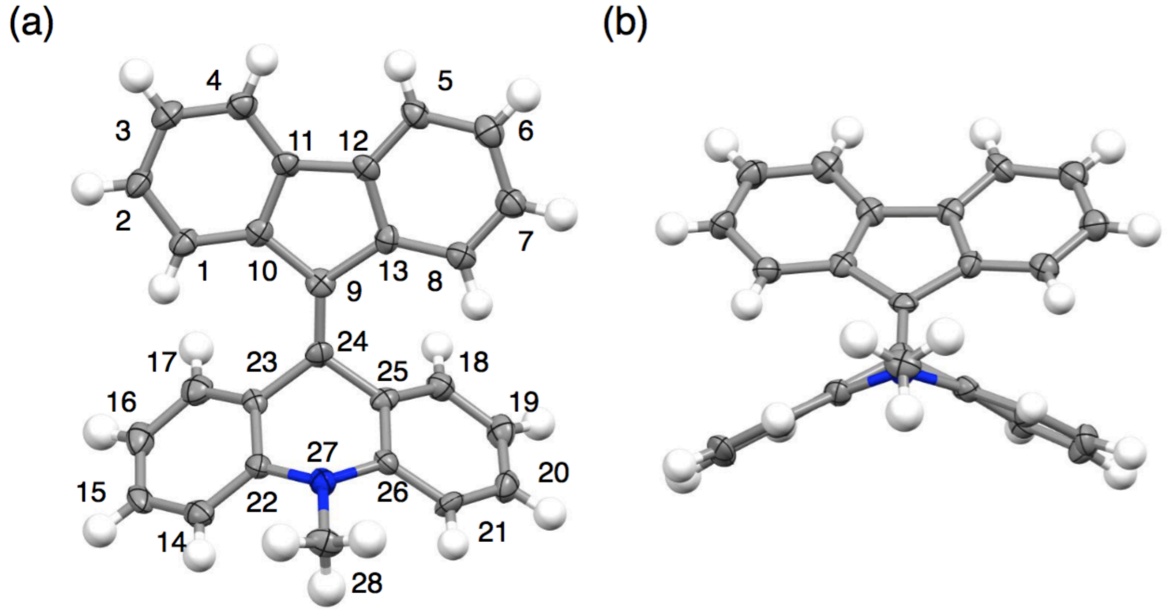

Figure S2. Crystal structure of **6a**, **Polymorph 1** in (a) top view and (b) side view. **Polymorph 1** contains two inequivalent molecular structures. One of them is shown here.

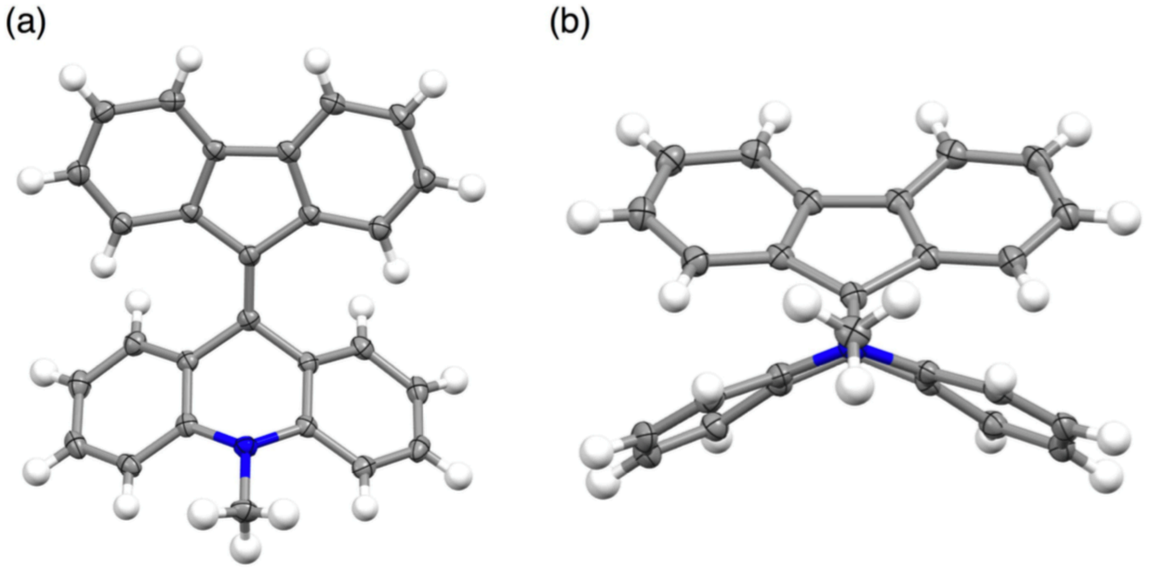

Figure S3. Crystal structure of **6a**, **Polymorph 2** in (a) top view and (b) side view.

Table S1. Crystal Data Collection Parameters for **5**.

Formula	$C_{26}H_{17}N$
Formula Weight	343.41
Crystal System	Orthorhombic
Space Group	*Pbca*
R, R_w (I>2σ(I))	0.0336, 0.1049
R1, wR2 (all data)	0.0368, 0.1091
GOF on F^2	0.929
a (Å)	8.2997(3)
b (Å)	33.2041(4)
c (Å)	12.4646(11)
α (º)	90
β (º)	90
γ (º)	90
V (Å^{-3})	3435.0(3)
Z	8
T (K)	123(2)
Crystal Size (mm)	0.28, 0.16, 0.15
D_{calcd} (g·cm^{-3})	1.328
$2\theta_{min}$, $2\theta_{max}$ (º)	8.88, 133.92
no. refl. Measured (unique)	3051
no. refl. measured (I>2σ(I))	2715
no parameters	312
λ (CuKα) (Å)	1.54187
CCDC deposition number	1457735

Table S2. Crystal Data Collection Parameters for **6a** (**Polymorph 1**).

Formula	$C_{27}H_{19}N$
Formula Weight	357.43
Crystal System	Monoclinic
Space Group	$P2_1$
R, R_w (I>2σ(I))	0.0628, 0.1697
R1, wR2 (all data)	0.0800, 0.2075
GOF on F^2	1.451
a (Å)	7.3384(2)
b (Å)	18.5774(6)
c (Å)	13.3299(4)
α (º)	90
β (º)	90.1024(18)
γ (º)	90
V (Å^{-3})	1817.24(9)
Z	4
T (K)	123(2)
Crystal Size (mm)	0.46, 0.30, 0.16
D_{calcd} (g·cm^{-3})	1.306
2θ_{min}, 2θ_{max} (º)	6.64, 133.98
no. refl. Measured (unique)	6277
no. refl. measured (I>2σ(I))	5264
no parameters	508
λ (CuKα) (Å)	1.54187
CCDC deposition number	1457737

Table S3. Crystal Data Collection Parameters for **6a** (**Polymorph 2**).

Formula	$C_{27}H_{19}N$
Formula Weight	357.43
Crystal System	Monoclinic
Space Group	$P2_1/a$
R, R_w (I>2σ(I))	0.0360, 0.1242
R1, wR2 (all data)	0.0403, 0.1303
GOF on F^2	1.120
a (Å)	8.5137(2)
b (Å)	21.9630(6)
c (Å)	9.8776(3)
α (º)	90
β (º)	104.4061(17)
γ (º)	90
V (Å^{-3})	1788.90(8)
Z	4
T (K)	123(2)
Crystal Size (mm)	0.30, 0.27, 0.24
D_{calcd} (g·cm^{-3})	1.327
2θ_{min}, 2θ_{max} (º)	8.06, 134.00
no. refl. Measured (unique)	3190
no. refl. measured (I>2σ(I))	2859
no parameters	330
λ (CuKα) (Å)	1.54187
CCDC deposition number	1457736

Table S4. Distances and angles of **6a** (**Polymorph 1**, **Polymorph 2** and DFT) and dimethylfluorenylidene-xanthone (Me_2FX)[1] for folded and twisted structures.

Bonds (Å) and Angles (°)	**Polymorph 1** (molecule 1)	**Polymorph 1** (molecule 2)	**Polymorph 2**	DFT (folded)	DFT (twisted)	Me_2FX (folded)	Me_2FX (twisted)
C9–C24	1.362(6)	1.391(6)	1.362(2)	1.373	1.412	1.352(5)	1.400(2)
C9–C10	1.489(5)	1.486(6)	1.500(2)	1.498	1.472	1.500(6)	1.477(2)
C9–C13	1.501(5)	1.489(6)	1.497(2)	1.498	1.469	1.496(6)	1.473(3)
C24–C23	1.493(5)	1.461(5)	1.478(2)	1.483	1.461	1.498(7)	1.466(2)
C24–C25	1.476(5)	1.461(5)	1.481(2)	1.483	1.460	1.481(6)	1.464(2)
C15–C16, C17–C23, C23–C22, and C22–C14	1.391(8), 1.392(7), 1.419(6), 1.401(6)	1.401(6), 1.398(6), 1.409(6), 1.407(5)	1.388(2), 1.396(2), 1.410(2), 1.400(2)	1.398, 1.402, 1.419, 1.405	1.403, 1.416, 1.424, 1.413	1.375(9), 1.392(7), 1.378(7), 1.389(8)	1.387(4), 1.403(2), 1.397(3), 1.388(3)
C14–C15 and C16–C17	1.384(7), 1.388(7)	1.390(6), 1.372(5)	1.387(2), 1.389(2)	1.393, 1.392	1.385, 1.382	1.386(9), 1.387(8)	1.363(3), 1.362(3)
Average of C14–C15, C15–C16, C16–C17, C17–C23, C23–C22, and C22–C14	1.396(7)	1.396(6)	1.395(2)	1.402	1.404	1.385(8)	1.383(3)
Dihedral angle C14C15C16C17C23C22–C18C19C20C21C26C25	52.00	52.63	50.29	48.22	13.11	44.50	6.02
Torsion angle C10–C13–C25–C23	−2.6(2)	1.2(2)	0.43(6)	0.00	43.93	−1.9(2)	40.39(8)
Distance between nitrogen and a plane N27···C22C26C28	0.216	0.245	0.230	0.183	0.000	–	–

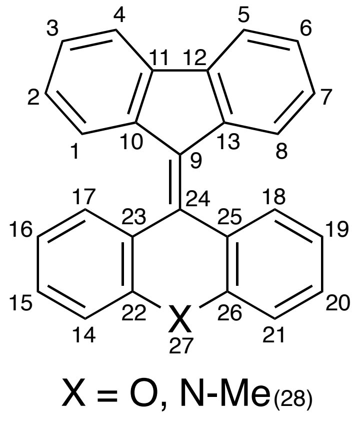

3. Light Absorption and Photoluminescence Spectra

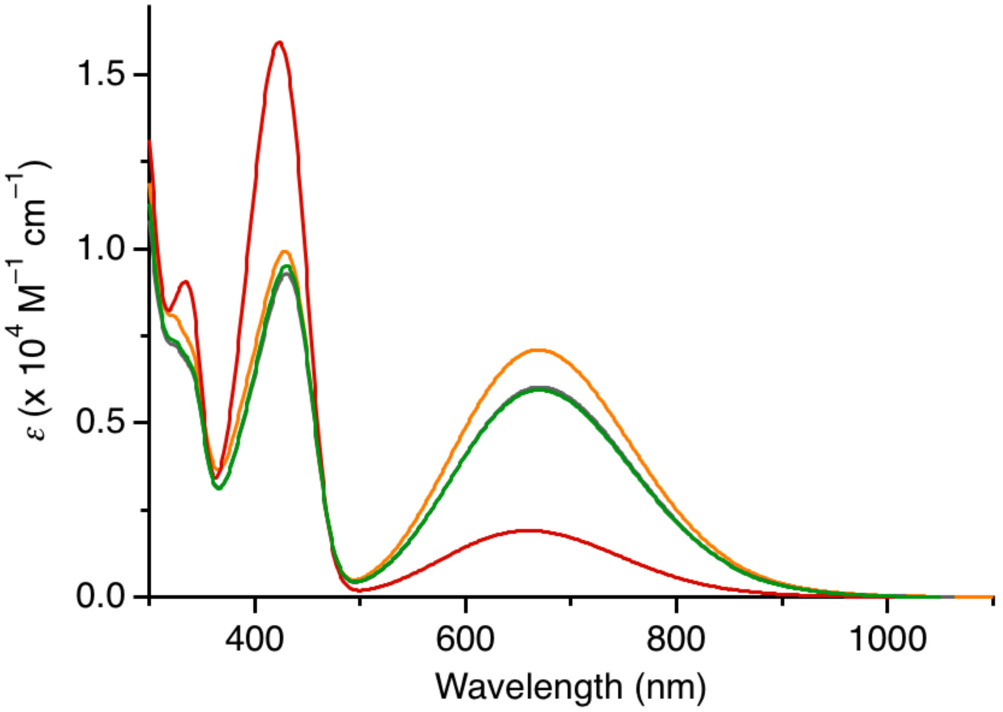

Figure S4. Light absorption spectra of FAs: **6a** (red), **6b** (orange), **6c** (gray) and **6d** (green) in CH_2Cl_2.

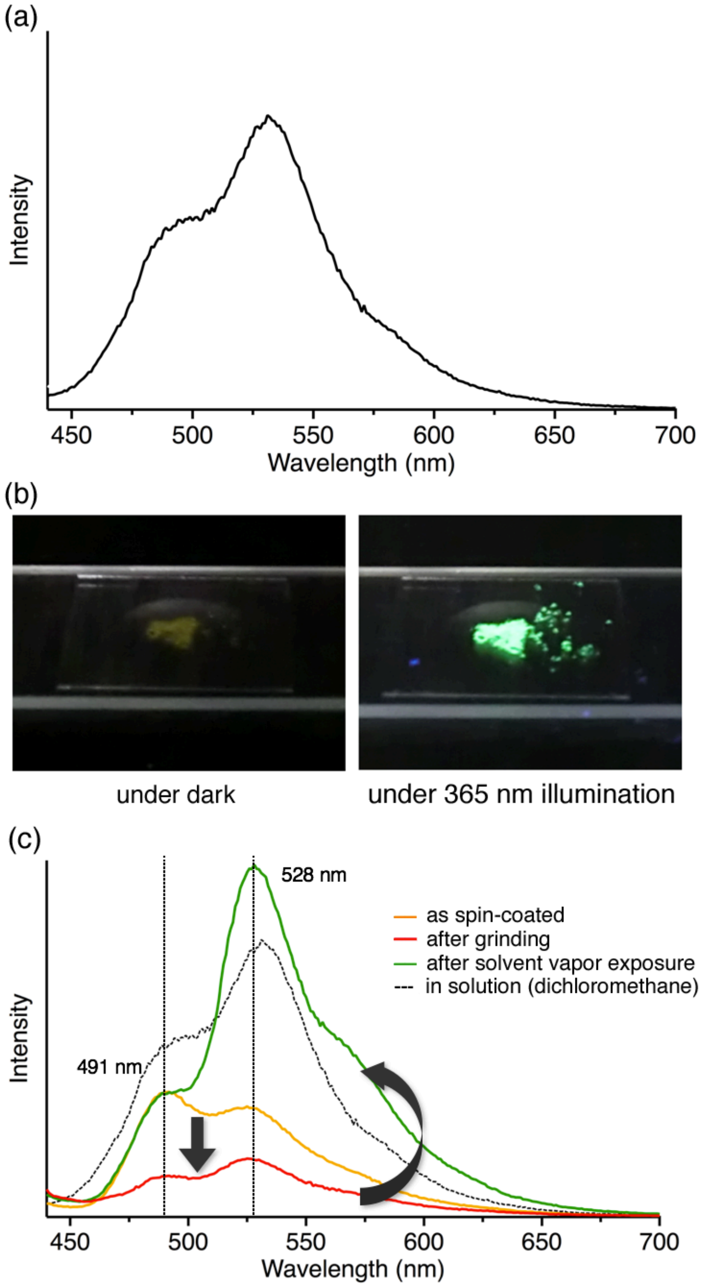

Figure S5. Luminescent property of **6a**. (a) Photoluminescence spectrum of **6a** in solution state (CH_2Cl_2). Excitation wavelength was 420 nm. No emission was detected by an excitation at 680 nm. (b) Solid state fluorescence of **6a** under 365 nm illumination. (c) Photoluminescence spectra of the solid samples of **6a**, as spin-coated, after grinding and solvent (chloroform) vapor exposure. A chlorobenzene solution of **6a** (10 mg/mL) was spin-coated on a quartz substrate. Excitation: 420 nm. Photoluminescence of the spin-coated sample exhibited larger emission at 491 nm than at 528 nm. After grinding, emission intensity decreased. After fuming, emission intensity became large, especially at 528 nm.

4. DFT Studies

Table S5. Calculated energetic levels of **6a-d** at B3LYP/6-31G(d) levels.

	**6a** folded/ twisted	**6b** folded/ twisted	**6c** folded/ twisted	**6d** folded/ twisted
**Gibbs Energy (Hartree)**	–1094.750232/ –1094.750100	–1134.033065/ –1134.038124	–1212.608503/ –1212.613295	–1369.754788/ –1369.758959
**$\Delta G_{\text{folded–twisted}}$ (kcal/mol)**	–0.0828	3.17	3.01	2.62
HOMO (eV)	–1.837/–2.316	–1.820/–2.311	–1.810/–2.309	–1.812/–2.310
LUMO (eV)	–5.219/–4.588	–5.169/–4.561	–5.138/–4.558	–5.136/–4.558
HOMO-LUMO gap (eV)	3.382/2.273	3.349/2.250	3.328/2.249	3.325/2.248

HOMO and LUMO levels among each folded structure and each twisted structure are similar, indicating that electronic effects of alkyl chains almost equal each other.

Table S6. Simulated light absorption wavelengths and oscillator strengths of **6a-d**. The calculations were carried out at B3LYP/6-31G(d) levels. The calculation data over 300 nm are listed. Intense absorption bands indicating >0.1 oscillator strengths were highlighted.

	**6a**		**6b**		**6c**		**6d**	
	folded	twisted	folded	twisted	folded	twisted	folded	twisted
Wavelength (nm), Oscillator Strength	**430.74, 0.4001**	**624.95, 0.4209**	**435.39, 0.4070**	**633.94, 0.4142**	**438.14, 0.4205**	**634.69, 0.4250**	**438.47, 0.4297**	**634.87, 0.4305**
	381.67, 0.0030	474.88, 0.0007	381.11, 0.0029	476.99, 0.0004	381.09, 0.0028	476.99, 0.0004	381.35, 0.0027	476.86, 0.0003
	**344.71, 0.1708**	393.31, 0.0230	**345.03, 0.1640**	392.58, 0.0289	**345.16, 0.1514**	393.14, 0.0318	**345.53, 0.1471**	393.19, 0.0327
	318.76, 0.0086	375.97, 0.0039	322.02, 0.0101	381.93, 0.0030	324.09, 0.0118	382.22, 0.0030	324.37, 0.0126	382.35, 0.0031
	307.84, 0.0395	364.25, 0.0538	308.24, 0.0280	365.63, 0.0604	309.28, 0.0153	365.76, 0.0620	309.32, 0.0142	365.83, 0.0627
		343.89, 0.0186		345.07, 0.0181		345.11, 0.0179		345.06, 0.0179
		328.48, 0.0236		331.00, 0.0243		331.06, 0.0237		331.14, 0.0233
		**305.44, 0.1235**		**304.48, 0.1101**		**304.53, 0.1070**		**304.53, 0.1050**

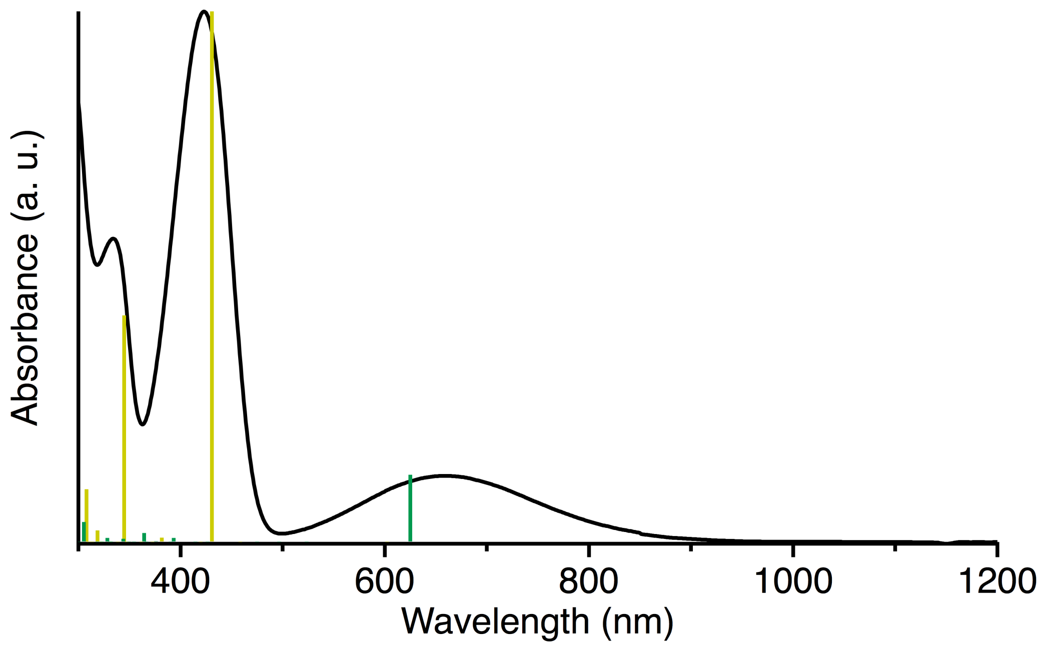

Figure S6. Simulation of light absorption bands of folded (yellow) and twisted (green) conformers of **6a** in CH_2Cl_2 (longitudinal rods) at the B3LYP/6-31G(d) level. Black line: experimental light absorption spectrum of **6a** in CH_2Cl_2.

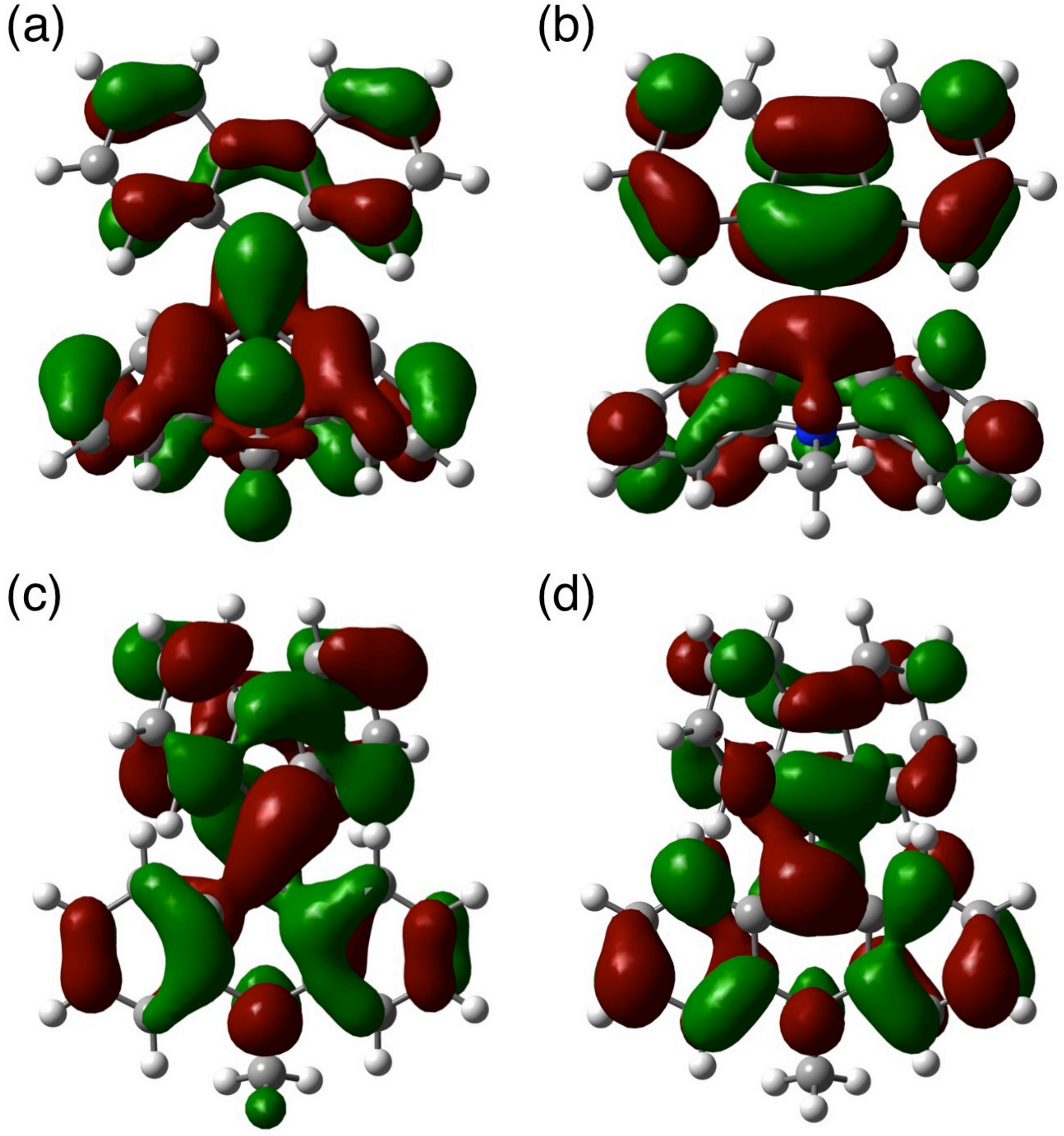

Figure S7. HOMO and LUMO structures of **6a**. (a) HOMO of folded conformer, (b) LUMO of folded conformer, (c) HOMO of twisted conformer, and (d) LUMO of twisted conformer are shown.

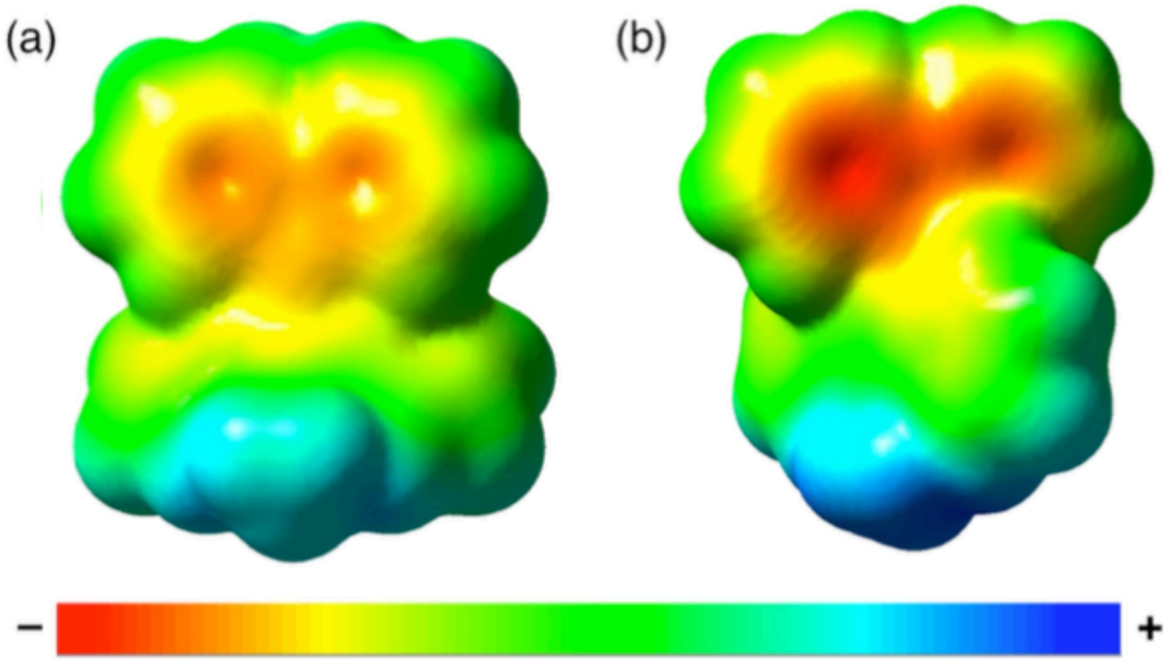

Figure S8. Electrostatic potential maps of **6a** in (a) the folded conformer and (b) in the twisted conformer.

5. Thermal Properties

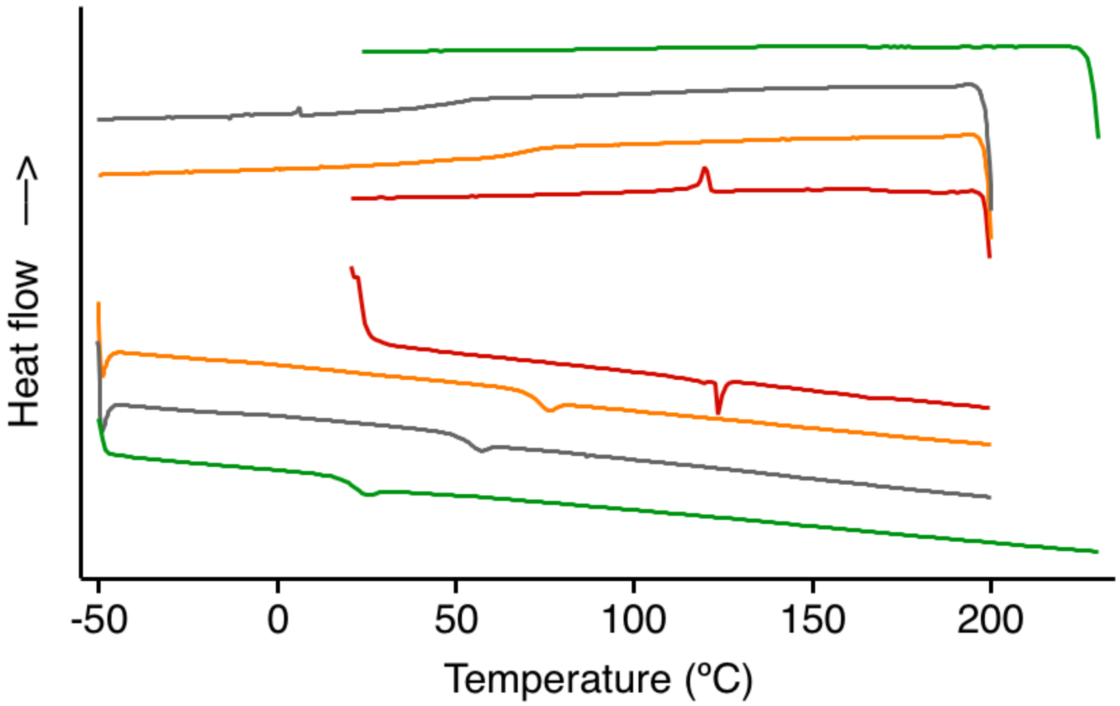

Figure S9. Differential scanning calorimetry (DSC) data of FAs. **6a** (reprecipitated) (red),**6b** (sublimated) (orange), **6c** (sublimated) (gray), and **6d** (reprecipitated) (green) were displayed. The first scans of heating and cooling processes for each FA are omitted. The second heating and followed cooling processes are in this figure. Half of under scans correspond to heating and the others are cooling processes. The sharp peaks found in **6a** data would indicate crystalline phase transition (m.p. of **6a**: 261.4–262.6 ºC). For the other FAs (**6b-d**), glass transitions were observed around 30–80 ºC.

6. Powder X-ray Diffraction Analysis

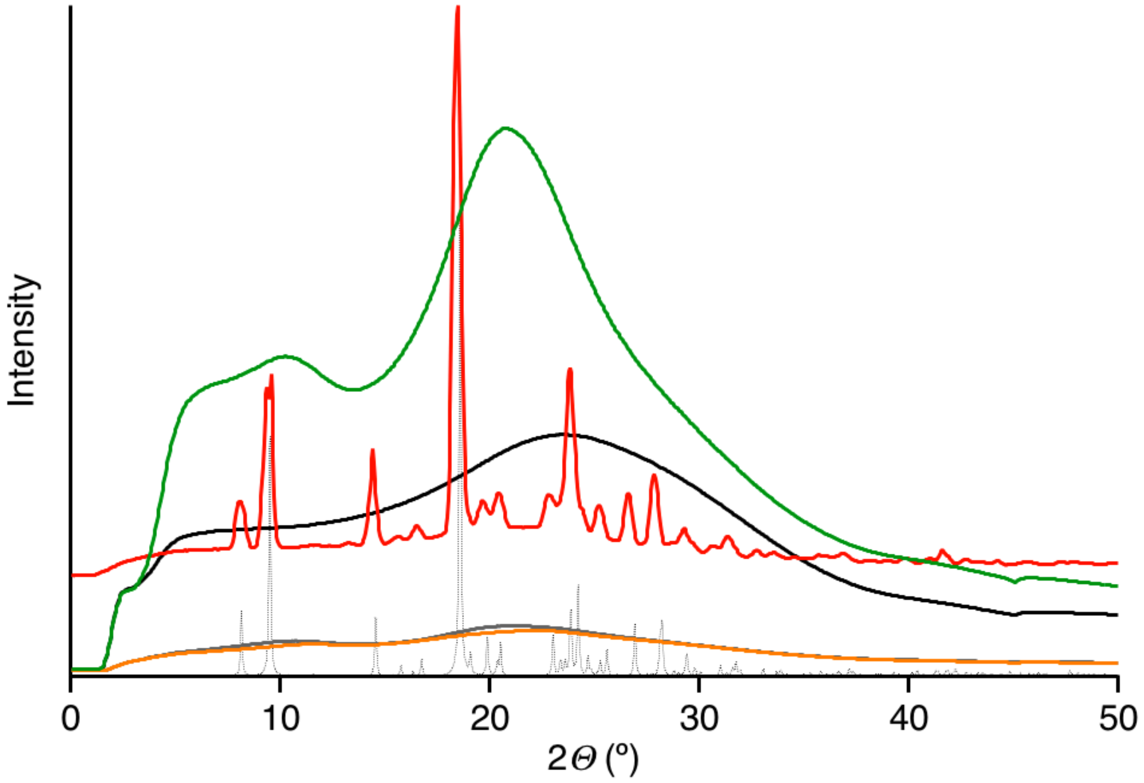

Figure S10. X-ray diffraction analysis of FA powder. **6a** (red), **6b** (orange), **6c** (gray), **6d** (green), blank tube (black) and simulated pattern from **6a** crystal data in Polymorph 1 (dot line) are displayed.

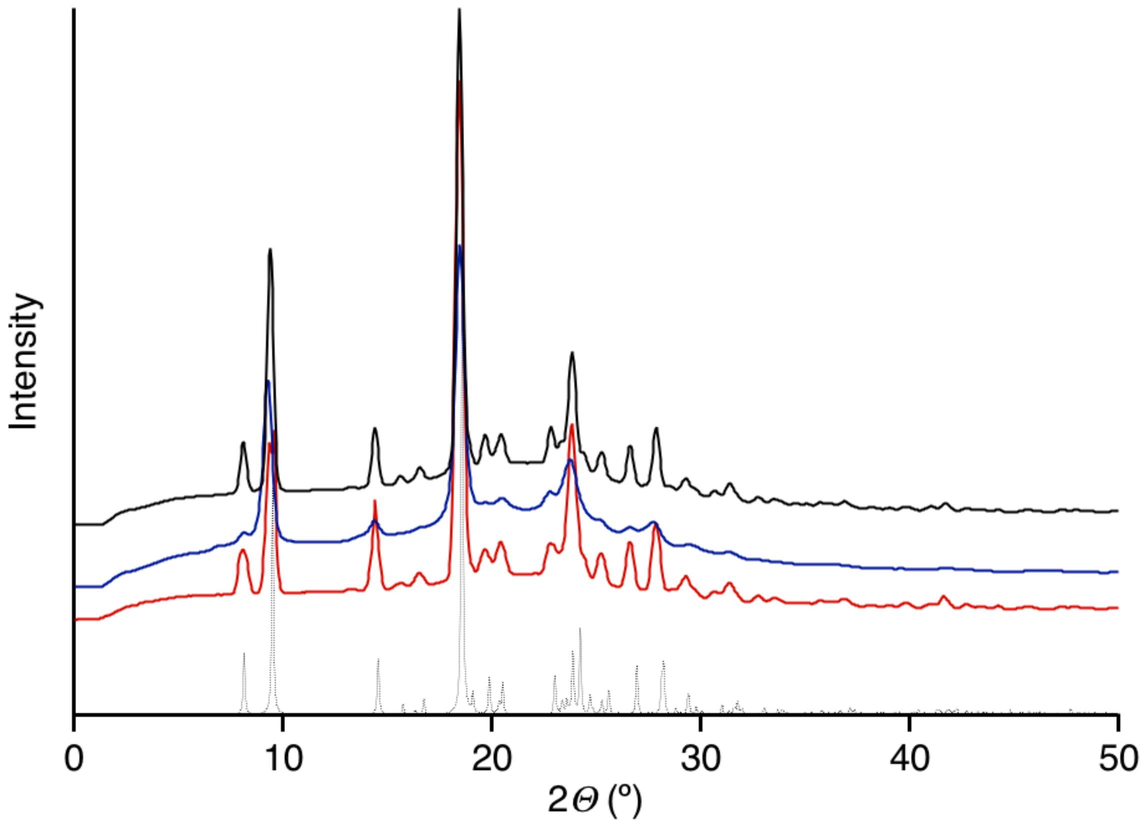

Figure S11. Powder XRD patterns of **6a** as reprecipitated (red), ground (blue), and solvent exposed (black) samples as well as simulated XRD pattern from **6a** crystal data in Polymorph 1 (dot line).

7. IR Spectra before and after grinding of 6a

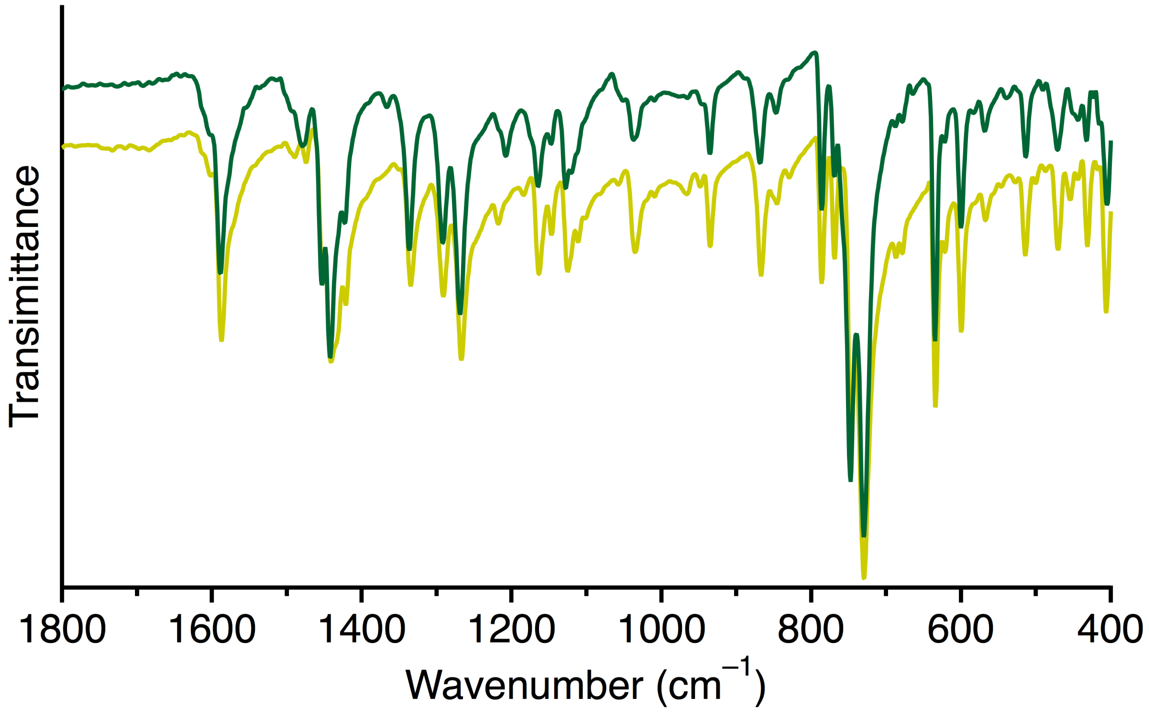

Figure S12. IR spectra before (greenish yellow line) and after (green line) grinding of the mechanochromic compound **6a**. These spectra are similar each other, but contains small difference. For example, there is difference around 1200 cm^{-1}. The C=C stretching vibration normally appear around 1500 cm^{-1}, where we expected spectral change for the C=C stretching vibration of the central C=C bond. However, it was overlapped with those of benzene rings. The C=C stretching vibration absorption at the fingerprint region from 700 to 1000 cm^{-1} was not obvious due to tetra-substitution of the alkene.

8. High-Pressure X-ray Single Crystal Analysis

High-pressure X-ray single crystal analysis was performed by a microfocused X-ray generator (MicroMax-007; Rigaku) with a Mo rotating target (λ = 0.7107 Å, 50 kV, 24 mA), confocal mirror optics (Varimax-Mo; Rigaku) and an imaging plate X-ray diffractometer (R-AXIS VI^{++}; Rigaku). Pressure was generated by using diamond–anvil cells with conically supported diamond anvils having half opening angle of 40°. Two single crystals of **6a, polymorph 2** are loaded in the cell with a mixture of MeOH and EtOH (v/v = 4/1) as a pressure medium. A small ruby crystal was also put into the cell, and its fluorescence was used to measure the pressure inside.[2]

Photographs of the crystals under respective pressures are shown in Figure S14. One crystal was broken into small pieces (one at the lower left, darker color). Color of the crystal was changed from greenish pale yellow to reddish brown under high pressure.

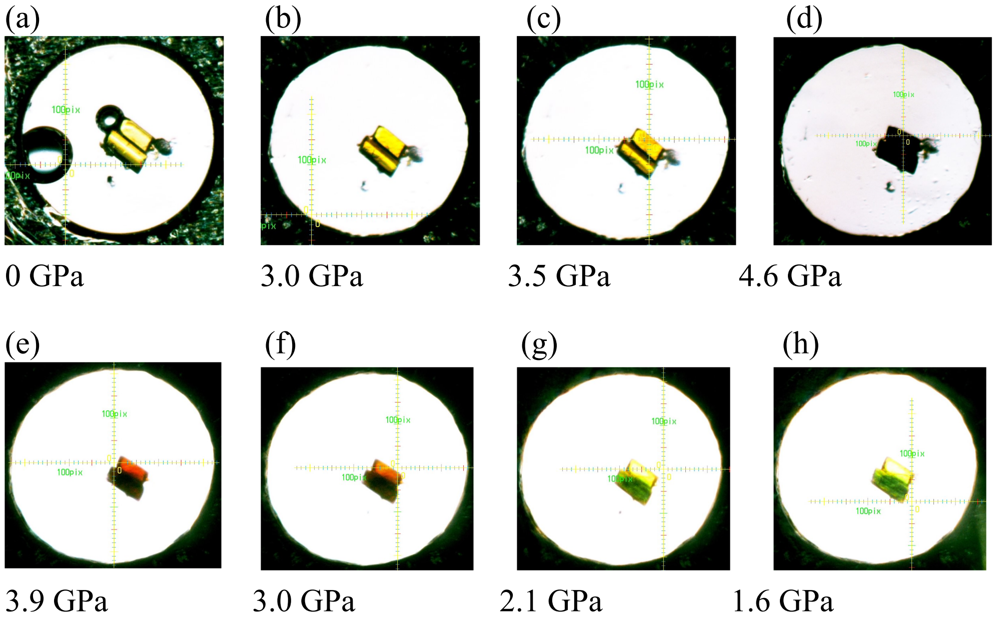

Figure S13 Photographs of the crystal under high pressure. (a) 0 GPa. (b) 3.0 GPa. (c) 3.5 GPa. (d) 4.6 GPa (The exposure condition was different from others). After the collection of X-ray diffraction, some photos were taken under lower pressure, (e) 3.9 GPa, (f) 3.0 GPa, (g) 2.1 GPa, (h) 1.6 GPa. Scale: 0.3 x 0.3 mm.

X-ray diffraction images were collected under 4.6–3.9 GPa (Figure S14d and e). Attenuation corrections for diamond anvils were applied, where incident and diffracted x-ray path lengths were taken into account. Analysis was performed using the SHELXL97 program on the Yadokari-software. The positional and thermal parameters were refined by a full-matrix least-squares method.

(a) (b)

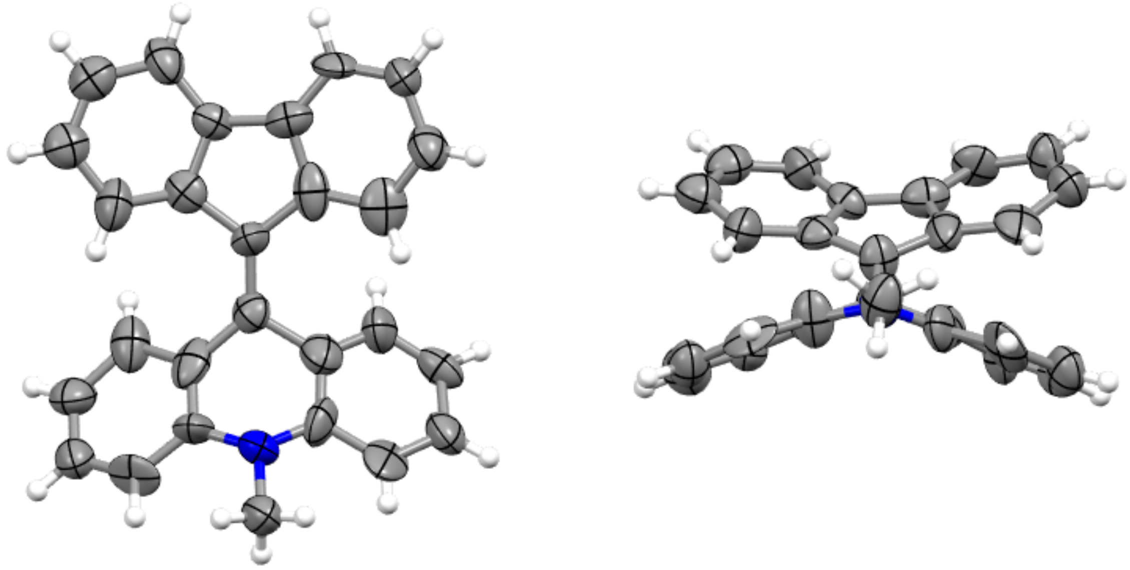

Figure S14. Crystal structure of compound **6a** under high pressure (4.6–3.9 GPa) in (a) top view and (b) side view.

Table S7. Crystal Data Collection Parameters for **6a** under high pressure.

Formula	$C_{27}H_{19}N$
Formula Weight	357.43
Crystal System	Monoclinic
Space Group	$P2_1/a$
R, R_w (I>2σ(I))	0.1217, 0.3147
R1, wR2 (all data)	0.1219, 0.3149
GOF on F^2	1.838
a (Å)	7.379(6)
b (Å)	22.716(16)
c (Å)	9.381(7)
α (º)	90
β (º)	111.14(2)
γ (º)	90
V (Å^{-3})	1466.51(10)
Z	4
T (K)	293(2)
D_{calcd} (g·cm^{-3})	1.619
2θ_{min}, 2θ_{max} (º)	4.66, 46.49
no. refl. Measured (unique)	623
no. refl. measured (I>2σ(I))	621
no parameters	254
λ (MoKα) (Å)	0.710700
CCDC deposition number	1457738

9. Electrochemical Studies

In solutions of FAs, folded and twisted conformers are in equilibrium. The electrochemical reactions of FAs should be described by considering the equilibrium. The analysis of equilibrated electrochemical reactions were carried out by referring the previous literature.[3]

According to the literature, the square scheme can be written as Figure S9. DFT calculations gave the results that the radical anions and radical cations preferentially form X-type conformer rather than V-type conformer. Accordingly, the electrochemical reduction reactions of FA derivatives are denoted as the following:

$$E_{K\mathrm{red}}^{\mathrm{o}} = E_{\mathrm{Xred}}^{\mathrm{o}} - (RT/F)\ \ln[(1 + K_1^{-1})/(1 + K_{2\mathrm{red}}^{-1})] \quad (1)$$

$$K_1 = [\mathbf{X}]/[\mathbf{V}],\ K_{2\mathrm{red}} = [\mathbf{X}^{\bullet-}]/[\mathbf{V}^{\bullet-}] >> 1 \quad (2)$$

Equation (1), (2) gave (T = 298 [K]):

$$E_{K\mathrm{red}}^{\mathrm{o}} \approx E_{\mathrm{Xred}}^{\mathrm{o}} - (RT/F)\ \ln[1 + K_1^{-1}] = E_{\mathrm{Xred}}^{\mathrm{o}} - 0.0257\ \ln[1 + K_1^{-1}] \quad (3)$$

The $E_{K\mathrm{red}}^{\mathrm{o}}$ value is observable in CV. Here, DFT study estimated the K_1^{-1} value of **6a** as 1.1 ([**V**]/[**X**] = 10/8.7) in CH_2Cl_2 solution. Consequently, $E_{K\mathrm{red}}^{\mathrm{o}} \approx E_{\mathrm{Xred}}^{\mathrm{o}}$, suggesting the first reduction wave in CV measurement represented the reduction potential of X-type conformer. As is the same to oxidation step (Figure S9-b), the electrochemical reaction is described as (T = 298 [K]):

$$E_{K\mathrm{ox}}^{\mathrm{o}} = E_{\mathrm{Xox}}^{\mathrm{o}} + (RT/F)\ln[(1 + K_{-1})/(1 + K_{2\mathrm{ox}})] \quad (4)$$

$$K_{2\mathrm{ox}} = [\mathbf{V}^{\bullet+}]/[\mathbf{X}^{\bullet+}] \approx 0,\ K_{-1} = [\mathbf{V}]/[\mathbf{X}] \quad (5)$$

From (4) and (5):

$$E_{K\mathrm{ox}}^{\mathrm{o}} \approx E_{\mathrm{Xox}}^{\mathrm{o}} + (RT/F)\ \ln[1 + K_{-1}] = E_{\mathrm{Xox}}^{\mathrm{o}} + 0.0257\ \ln[1 + K_{-1}] \quad (6)$$

The K_{-1} value of **6a** is also 1.1, and therefore $E_{K\mathrm{ox}}^{\mathrm{o}} \approx E_{\mathrm{Xox}}^{\mathrm{o}}$. The $E_{K\mathrm{ox}}^{\mathrm{o}}$ is also observable value in CV measurement. This theoretical investigation clearly demonstrated that the CV charts of FA independently displayed reduction and oxidation of twisted conformer in the first reduction and oxidation waves. The equilibrium between folded and twisted conformers influences to the electrochemical reactions, however the degrees are quite small in this case. This simple picture is derived from the highly energetic difference between radical folded and twisted conformers.

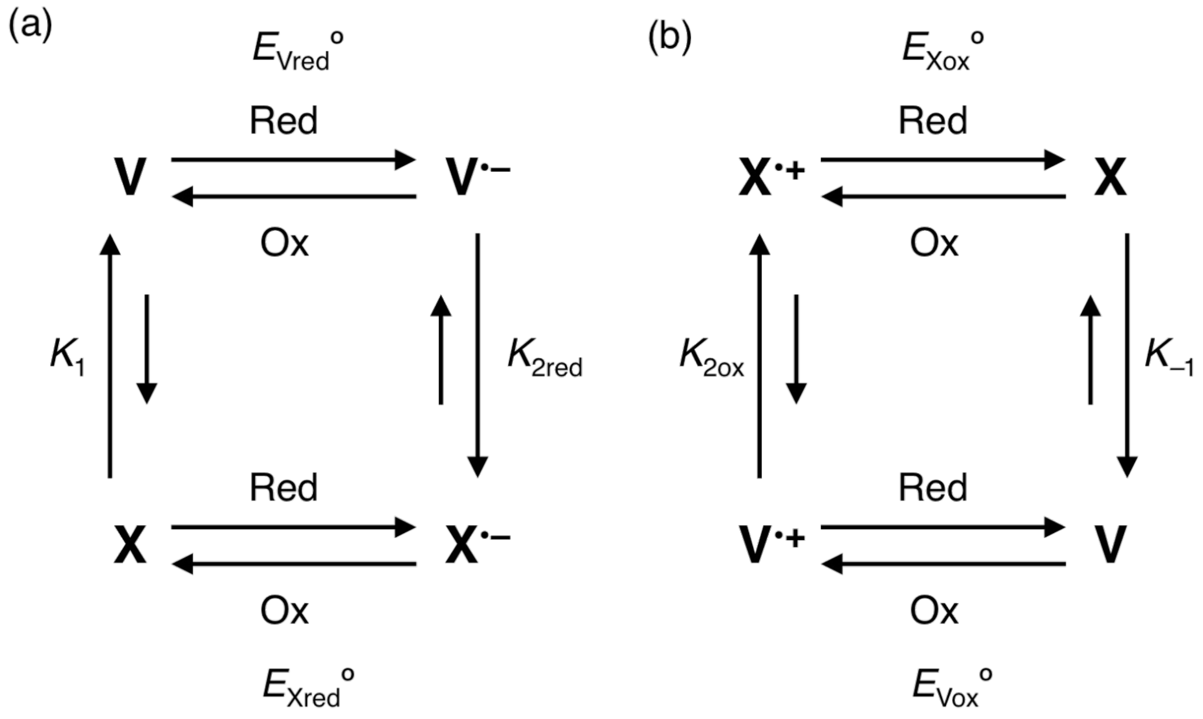

Figure S15. The four-member square scheme of cyclic voltammetry. These are the models of (a) the first reduction and (b) the first oxidation of Me-FA.

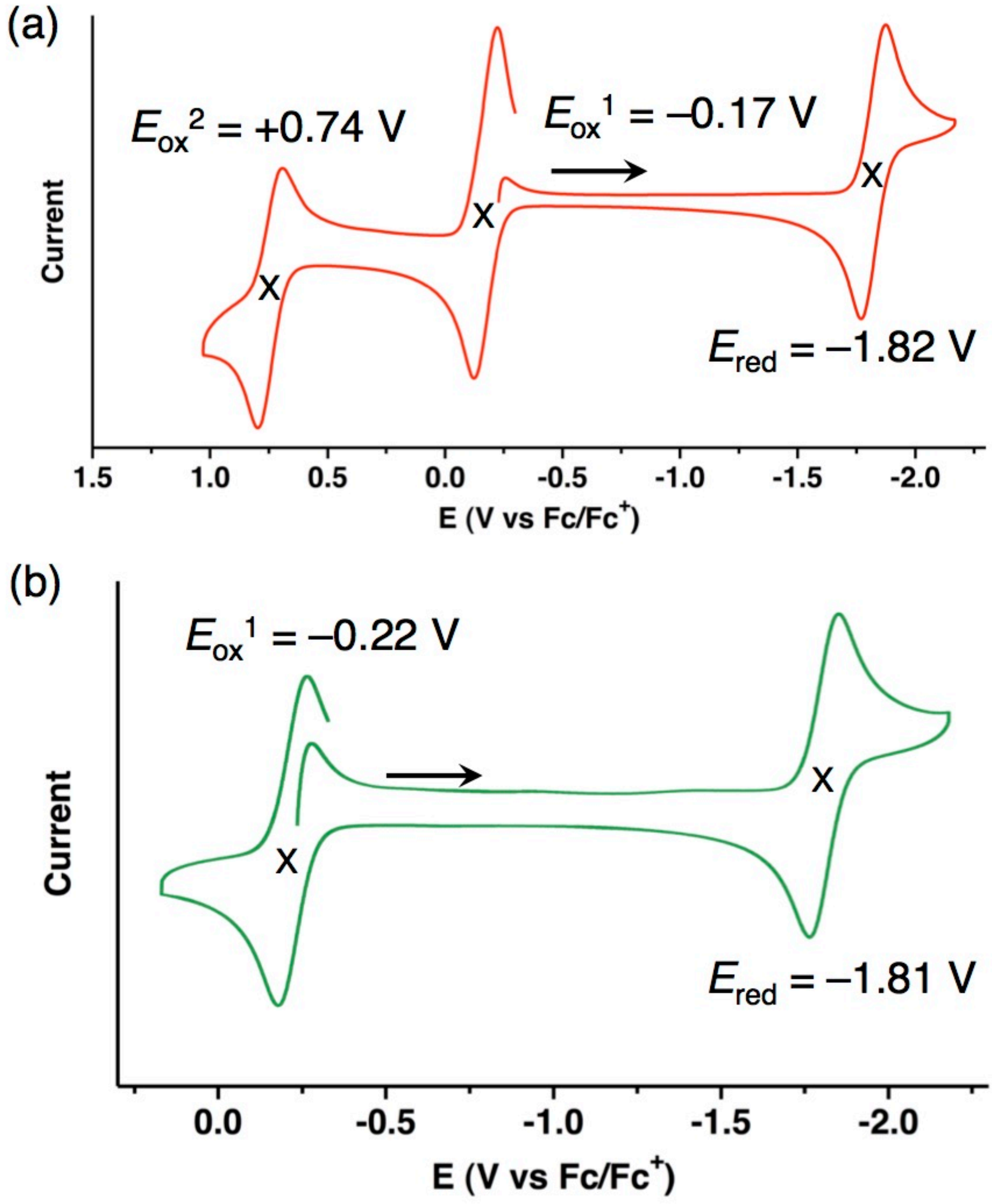

Figure S16. Cyclic voltammogram of (a) **6a** and (b) **6d**. It was measured in CH_2Cl_2 under argon atmosphere with glassy-carbon, platinum and Ag/Ag^+ for working, counter and reference electrodes, respectively. $TBAPF_6$ was added in the solution as an electrolyte.

10. Carrier Transport Properties

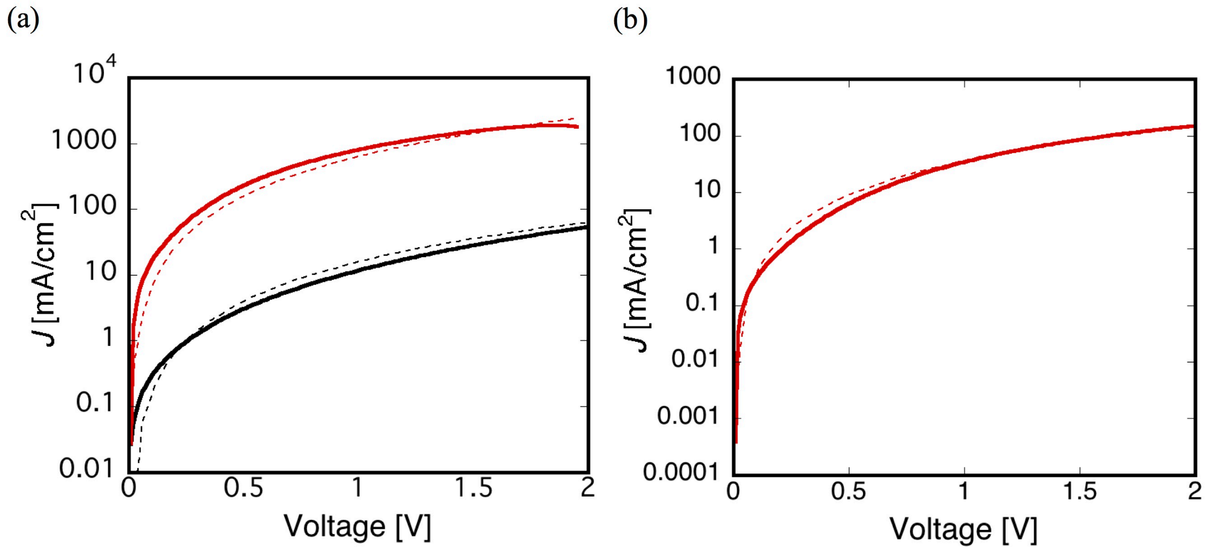

Figure S17. Space charge limited current (SCLC) chart for hole and electron transport were collected. Current density was plotted against voltage for (a) hole mobility and (b) electron mobility. The spectra of **6a** and **6d** are drawn in black and red, respectively. Device configurations of the hole-only and electron-only devices are glass/ITO/PEDOT:PSS/**6a,d**/MoO_3/Al and glass/Al/**6a,d**/LiF/Al, respectively.

The equation to calculate the mobilities is followed:

$$J = \frac{9}{8}\varepsilon_0 \cdot \varepsilon_r \mu \frac{V^2}{L^3}$$

where J is the current density, ε_0 is the permittivity of free space, ε_r is the relative permittivity of the material, μ is the mobility, V is the effective voltage, and L is the thickness of the active layer. The hole mobilities of **6a** and **6d** are calculated as 3.0×10^{-5} and 8.4×10^{-4} cm^2/Vs, respectively, and the electron mobility of **6d** is 4.8×10^{-5} cm^2/Vs.

11. References

1. "Polymorphism versus thermochromism: interrelation of color and conformation in overcrowded bistricyclic aromatic enes", P. U. Biedermann, J. J. Stezowski, I. Agranat, *Chem. Eur. J.* **2006**, *12*, 3345–3354.

2. "Calibration of the pressure dependence of the *R*1 ruby fluorescence line to 195 kbar", E. Laviron, L. Roullier, *J. Electroanal. Chem.* **1985**, *186*, 1–15.

3. "The square scheme with the electrochemical reactions at equilibrium A study by diffusion or thin layer cyclic voltammetry and by scanning potential coulometry", G. J. Piermarini, S. Block, J. D. Barnett, R. A. Forman, *J. Appl. Phys.* **1975**, *46*, 2774–2780.
